# Therapeutic Hypothermia Alleviates Hydrocephalus and Neurological Dysfunction Post Intraventricular Hemorrhage by Enhancing Drainage of Glymphatic‐Meningeal Lymphatic‐Deep Cervical Lymphatic System

**DOI:** 10.1002/cns.70740

**Published:** 2026-01-24

**Authors:** Yijian Yang, Qian Ouyang, Yaxin Sun, Aijun Liang, Junqiang Wang, Kaiyue Wang, Yexin Yuan, Zhikun Liu, Jun Huang, Zhiping Zhang, Libin Wang, Yang Yuan, Cheng Wang, Zhangjie Su, Qinghua Zhang, Liyang Zhang, Gelei Xiao

**Affiliations:** ^1^ Department of Neurosurgery, Xiangya Hospital Central South University Changsha Hunan China; ^2^ Diagnosis and Treatment Center for Hydrocephalus, Xiangya Hospital Central South University Changsha Hunan China; ^3^ Hunan International Scientific and Technological Cooperation Base of Brain Tumor Research, Xiangya Hospital Central South University Changsha Hunan China; ^4^ National Clinical Research Center for Geriatric Disorders, Xiangya Hospital Central South University Changsha Hunan China; ^5^ Department of Neurosurgery, Zhuzhou Hospital Affiliated to Xiangya School of Medicine Central South University Zhuzhou Hunan China; ^6^ Department of Neurosurgery, Xiangya Hospital (Jiangxi), National Regional Center for Neurological Diseases Central South University Nanchang Jiangxi China; ^7^ Department of Neurosurgery Shenzhen Nanshan People's Hospital Shenzhen Guangdong China; ^8^ Department of Neurosurgery The Second Affiliated Hospital of Xinjiang Medical University Urumqi Xinjiang China; ^9^ Department of Neurosurgery Changsha Hospital of Traditional Chinese Medicine (Changsha Eighth Hospital) Changsha Hunan China; ^10^ Department of Neurosurgery, John Radcliffe Hospital Oxford University Hospitals NHS Foundation Trust Oxford UK; ^11^ Department of Neurosurgery General Hospital of Ningxia Medical University Yinchuan Ningxia China

**Keywords:** cerebrospinal fluid homeostasis, glymphatic‐meningeal lymphatic system‐deep cervical lymphatic system, hematoma clearance, hydrocephalus post intraventricular hemorrhage, neurological dysfunction, neurovascular unit, RTN3/AMPK/ERS, therapeutic hypothermia

## Abstract

**Aims:**

Intraventricular hemorrhage (IVH) has a high mortality and disability rate and poses significant clinical challenges, which often leads to hydrocephalus and neurological dysfunction. Emerging evidence implicates dysfunction of the glymphatic‐meningeal lymphatic‐deep cervical lymphatic system in the pathogenesis of post‐IVH hydrocephalus. Therapeutic hypothermia (TH) holds promise for mitigating these sequelae. This study investigates the potential of TH to ameliorate IVH‐induced hydrocephalus and neurological dysfunction, while elucidating its underlying mechanisms.

**Methods:**

Rat models of IVH are established by intraventricular injection of autologous blood. TH, deep cervical lymph nodes (dCLNs) ligation, and intraperitoneal injection of AMPK inhibitor are used to intervene.

**Results:**

In rat models of IVH, TH alleviates neurological dysfunction and attenuates hydrocephalus and pathology damage. TH protects physiological function and maintains normal structure of the glymphatic system. TH improves function and structure of the meningeal lymphatic system and promotes drainage of the deep cervical lymphatic system. TH upregulates RTN3, facilitates phosphorylation of AMPK, and suppresses ERS. All the above effects are reversed by ligation of dCLNs and AMPK inhibitor.

**Conclusion:**

TH alleviates hydrocephalus and neurological dysfunction post IVH by enhancing drainage of the glymphatic‐meningeal lymphatic‐deep cervical lymphatic system. The RTN3/AMPK/ERS pathway may be the proposed mechanism mediating this effect. TH is expected to become a novel therapeutic strategy for IVH.

## Graphical abstract

TH is expected to be a novel therapeutic strategy for hydrocephalus and neurological dysfunction post IVH. TH alleviates hydrocephalus post IVH by enhancing drainage of the glymphatic‐meningeal lymphatic‐deep cervical lymphatic system, which is the connection between the central nervous system and peripheral lymphatic system. Cold stress response caused by TH improves the function of the glymphatic‐meningeal lymphatic‐deep cervical lymphatic system by regulating RTN3/AMPK/ERS.

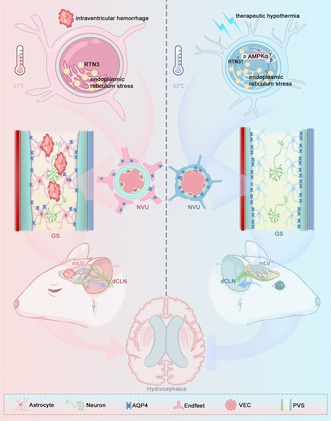

## 1 Introduction

Intraventricular hemorrhage (IVH) brings great threats to patients and seriously affects their safety and quality of life [1, 2] and has a high incidence at all ages and often causes hydrocephalus and neurological dysfunction [3, 4]. Hydrocephalus is defined as the abnormal accumulation of cerebrospinal fluid (CSF) in the ventricular system, with or without its dilation [5, 6]. In addition to headache caused by intracranial hypertension, it can also lead to a series of neurological dysfunctions due to compression of brain tissue [7, 8]. At present, patients with hydrocephalus post IVH are often treated by surgery in clinical work. Commonly used surgical procedures include ventriculoperitoneal shunt, endoscopic third ventriculostomy, and choroid plexus cauterization [9]. Although the surgical procedure has been strategically optimized, the problems caused by the surgery itself, such as postoperative infection, shunt blockage and rupture, and lifelong carrying of the shunt, will affect the patient's postoperative recovery. Therefore, it is of great practical significance to explore new therapeutic strategy and explain its mechanism.

The essential cause of hydrocephalus is the imbalance of CSF dynamic homeostasis, which is closely related to the intracranial fluid transport system. The glymphatic system (GS), which was discovered firstly in 2012 [10], is considered to exist in the perivascular space (PVS) [11]. The basic structure of the GS is the neurovascular unit (NVU), formed between the astrocyte endfeet surrounding the blood vessel and the capillary endothelial cells [12, 13]. Under the arterial pulsation‐driven CSF influx, it merges with the interstitial fluid through aquaporin‐4 (AQP4) on the endfeet membrane of astrocytes around the PVS [14]. Thus, it plays an important role in the removal of CSF and metabolic products and the recovery of neurological function. Then, it takes the metabolic waste and neurotoxicants produced by the brain tissue away through the meningeal lymphatic system, which was formed by meningeal lymphatic vessels (mLVs) [15]. Research on meningeal lymphatics began with Paolo Mascagni's description in 1787. Axel Key and Gustaf Retzius confirmed CSF drains to cervical lymph nodes in 1875 [16]. In 2015, meningeal lymphatic vessels (mLVs) were confirmed to drain CSF and macromolecules to deep cervical lymph nodes (dCLNs), revising earlier beliefs of a CNS lacking lymphatics [17]. Then, through step‐by‐step lymphatic drainage, it enters the deep cervical lymph nodes (dCLNs), and further introduces the metabolic waste in the brain cleared by the GS into the deep cervical lymphatic system, thereby filtering or neutralizing potentially harmful substances in the CSF [18]. Historically, studies since the 1980s indicated ~50% of CSF drains via dCLNs, establishing mLVs and dCLNs as critical pathways in CNS waste clearance and immune surveillance [19]. Thus, the cascade of sequential drainage steps of the glymphatic‐meningeal lymphatic‐deep cervical lymphatic system, which are functionally linked as a multi‐compartment clearance axis, plays a crucial role in maintaining CSF dynamic homeostasis in hydrocephalus post IVH.

As an auxiliary intervention, therapeutic hypothermia (TH), which refers to controlling the central temperature between 32°C and 34°C to activate the cold stress response and reduce cell metabolic rate, has been found to play a role in neuroprotective effect [20]. The concept that TH has brain protective function has been gradually accepted by neurosurgeons and tentatively applied to the treatment of severe traumatic brain injury (TBI) [21], subarachnoid hemorrhage [22], stroke [23] and other diseases. In order to explain the protective mechanism of TH on glymphatic‐meningeal lymphatic‐deep cervical lymphatic system at the molecular level, we look to the effector proteins of cold stress response. Reticulon‐3 (RTN3) is a cold stress response‐related protein that is upregulated after stimulation by cold stress response [24], and localized in endoplasmic reticulum (ER) especially in the brain. Studies have confirmed that the upregulation of RTN3 has neuroprotective effect on rats [25, 26]. The adenosine 5′ monophosphate‐activated protein kinase (AMPK) signaling pathway plays a key role in the regulation of cellular energy homeostasis. Studies have shown that mild hypothermia upregulates the phosphorylation of AMPK, thereby inhibiting the activation of NLRP3 inflammasome to alleviate early brain injury after subarachnoid hemorrhage [27, 28]. In recent years, studies have found that ERS plays an important role in a variety of neurological diseases, including IVH [29], neurodegenerative diseases [30], and TBI [31]. Accordingly, RTN3/AMPK/ERS may be a molecular mechanism to regulate the drainage of glymphatic‐meningeal lymphatic‐deep cervical lymphatic system, due to their distribution location, functional effects and causes of changes.

Based on the above contents, we established a rat model of IVH and adopt multidisciplinary experimental methods to explore and study: (1) TH can alleviate hydrocephalus and neurological dysfunction post IVH by enhancing drainage of glymphatic‐meningeal lymphatic‐deep cervical lymphatic system. (2) TH can protect glymphatic‐meningeal lymphatic‐deep cervical lymphatic system via inhibiting RTN3/AMPK/ERS pathway. The results will provide a theoretical basis for the clinical development of effective therapeutic strategy for the treatment of hydrocephalus post IVH, improve the treatment efficiency of patients with hydrocephalus post IVH, and bring greater clinical benefits to patients.

## 2 Methods

### 2.1 Experiment Design and Groups

The study included seven experiments, which were shown in Figure S1. The rats were randomly divided into the sham group (sham operation without autologous blood injection), IVH group (IVH), IVH + TH group (treatment with TH post IVH), IVH + TH + ligation group (dCLNs ligation before treatment with TH post IVH), and IVH + TH + inhibitor group (AMPK inhibitor Dorsomorphin injection after treatment with TH post IVH).

### 2.2 Animals and Ethics

The study included a total of 243 male Sprague Dawley rats weighing between 200 and 250 g (9.88% mortality and 219 rats survived and sacrificed for experiment finally). Details of the numbers of animal used in the experiment are provided in Table S1. These rats were obtained from Xiangya hospital of Central South University. A reverse 12‐h light/12‐h dark cycle environment was used to acclimate the rats, who were also provided with unlimited access to water and food. All procedures followed the guidelines of the Experimental Animal Ethics Committee, Xiangya hospital of Central South University (202503038). This study followed the Animal Research: Reporting of In Vivo Experiments guidelines for reporting animal experiments.

### 2.3 Intraventricular Hemorrhage Model

We established IVH rats' model by injecting autologous blood in right lateral ventricle as previously described [32]. Detailed establishment process can be seen in Appendix S1 and Figure S2A.

### 2.4 Therapeutic Hypothermia

According to relevant literature [27], we defined TH as 33°C ± 1°C. We kept the rectal temperature of rats within 33°C ± 1°C for 6 h by circularly transferring rats between heating pad and ice pad. Detailed steps can be seen in Appendix S1 and Figure S2B.

### 2.5 Deep Cervical Lymph Nodes Ligation

The ligation of dCLNs described previously [33], was performed 1 day before IVH. Detailed establishment process can be seen in Appendix S1 and Figure S2C.

### 2.6 Dorsomorphin Injection

Dorsomorphin (HY‐13418A, MCE, United States) is an inhibitor of AMPK and is dissolved in dimethyl sulfoxide and then diluted with saline (0.9% NaCl). The dosage and method of administering Dorsomorphin were consistent with previous studies [34], with a dose of 0.2 mg/kg injected through intraperitoneal once a day. It would be dosed postoperatively and continue to the seventh day in experiment 1 and the third day in other experiments.

### 2.7 Ablation of MLVs

According to relevant literature [35], we used the method of light irradiation ablation after visudyne injection into the cisterna magna. Detailed establishment process can be seen in Appendix S1.

### 2.8 Behavioral Test

We used modified neurologic severity score (mNSS); Corner turn test; Cylinder test; Open field test (OFT); Novel object recognition (NOR); and Morris water maze (MWM) to evaluate the neurological function of rats. Details are described in Appendix S1.

### 2.9 Magnetic Resonance Imaging

The MRI scanning was performed using a 3.0‐T scanner (Siemens, Germany). T2 sequence (slice thickness 1.2 mm; TR 2780 ms; TE 121 ms) was set during this experiment. T1 sequence (slice thickness 1.2 mm; TR 500 ms; TE 9.3 ms) was set during this experiment after injection of Gadopentetate Dimeglumine (Gd‐DTPA) (H10950231, Consun, China) in cisternal magna. Detailed steps can be seen in Appendix S1 and Figure S2D.

Brain section, dCLNs section, meninges preparation, histological staining and Transmission electron microscopy (TEM): Details are described in Supplementary Methods in Supplementary File.

### 2.10 Immunofluorescence (IF)

Details are described in Appendix S1. The primary antibodies used can be seen in Table S2.

### 2.11 Intracisternal Magna Injection and Inflow

The brain was removed and sectioned into four coronal brain sections at bregma +1.6 mm, −2.4 mm, −3.2 mm, and −4.0 mm with a thickness of 100 μm using a cryostat microtome [36]. Detailed steps can be seen in Appendix S1 and Figure S2D.

### 2.12 Intrahippocampal Injection and Outflow

The brain was removed and sectioned into four coronal brain sections at bregma −1.6 mm, −2.4 mm, −3.2 mm, and −4.0 mm with a thickness of 100 μm using a cryostat microtome [36]. Detailed steps can be seen in Appendix S1 and Figure S2A.

### 2.13 AQP4 Polarization Measurement

Initially, one method involved calculating AQP4 polarization from the IF of GFAP and AQP4. Specifically, a donut‐shaped region was drawn within 5 pixels from a capillary, encompassing the void surrounded by areas rich in DAPI and GFAP expression. The AQP4 polarization was calculated as AQP4 in the donut‐shaped/global mean. Alternatively, we assessed AQP4 polarization by analyzing the IF of CD31 and AQP4. Randomly, a line perpendicular to the vessel was chosen and recorded the fluorescence intensity along this line. AQP4 polarization was determined as the ratio of the peak AQP4 intensity/global mean. Moreover, AQP4 polarization could be defined as the ratio of AQP4 M23/AQP4 M1 in protein expression in WB.

### 2.14 Western Blot (WB)

WB was performed 3 days after IVH as described previously. The primary antibodies used can be seen in Table S3. A density analysis of the bands was performed using ImageJ.

### 2.15 Cell Culture

Rat astrocytes (KC9642, Kinlogix Biotech Co. Ltd., China) were cultured and passaged in complete medium for rat astrocytes (KC9642M, Kinlogix Biotech Co. Ltd., China). Mouse lymph node endothelial cells (CL‐0221, Procell Biotech Co. Ltd., China) were cultured and passaged in complete medium for mouse lymph node endothelial cells (CM‐0221, Procell Biotech Co. Ltd., China). After three subcultures, the cells were used for subsequent experiments. To construct an in vitro IVH model, an extensive literature search was conducted, in which each group of cells was treated with 30 U/mL thrombin (T4648, Sigma, USA) dissolved in 0.1% bovine serum albumin solution, followed by incubation for 6 h. The cells were divided into four groups: control, T (thrombin), T + TH, and T + TH + inhibitor (Dorsomorphin). Incubator temperature was adjusted to 33°C to simulate TH. The Dorsomorphin intervention concentration was 15 μM.

### 2.16 Apoptosis Assay

Apoptosis was assessed using the Annexin V‐FITC/PI cell apoptosis detection kit (MA0220‐2, meilunbio). Cells in the different experimental groups were trypsinized without EDTA, suspended in binding buffer, and incubated with Annexin V‐FITC and PI for 15 min. Flow cytometry (LongCyteC2060, CHALLEN BIO, China) was then performed to analyze the results.

### 2.17 Statistical Analysis

All data are presented as the mean ± SD, and differences between mean values were assessed by one‐way analysis of variance (ANOVA) or two‐way ANOVA and Tukey's multiple comparison test using GraphPad Prism (GraphPad Software, America). *p* values < 0.05 were considered significant.

## 3 Results

### 3.1 TH Alleviates Neurological Dysfunction Post IVH in Rats

**FIGURE 1:**
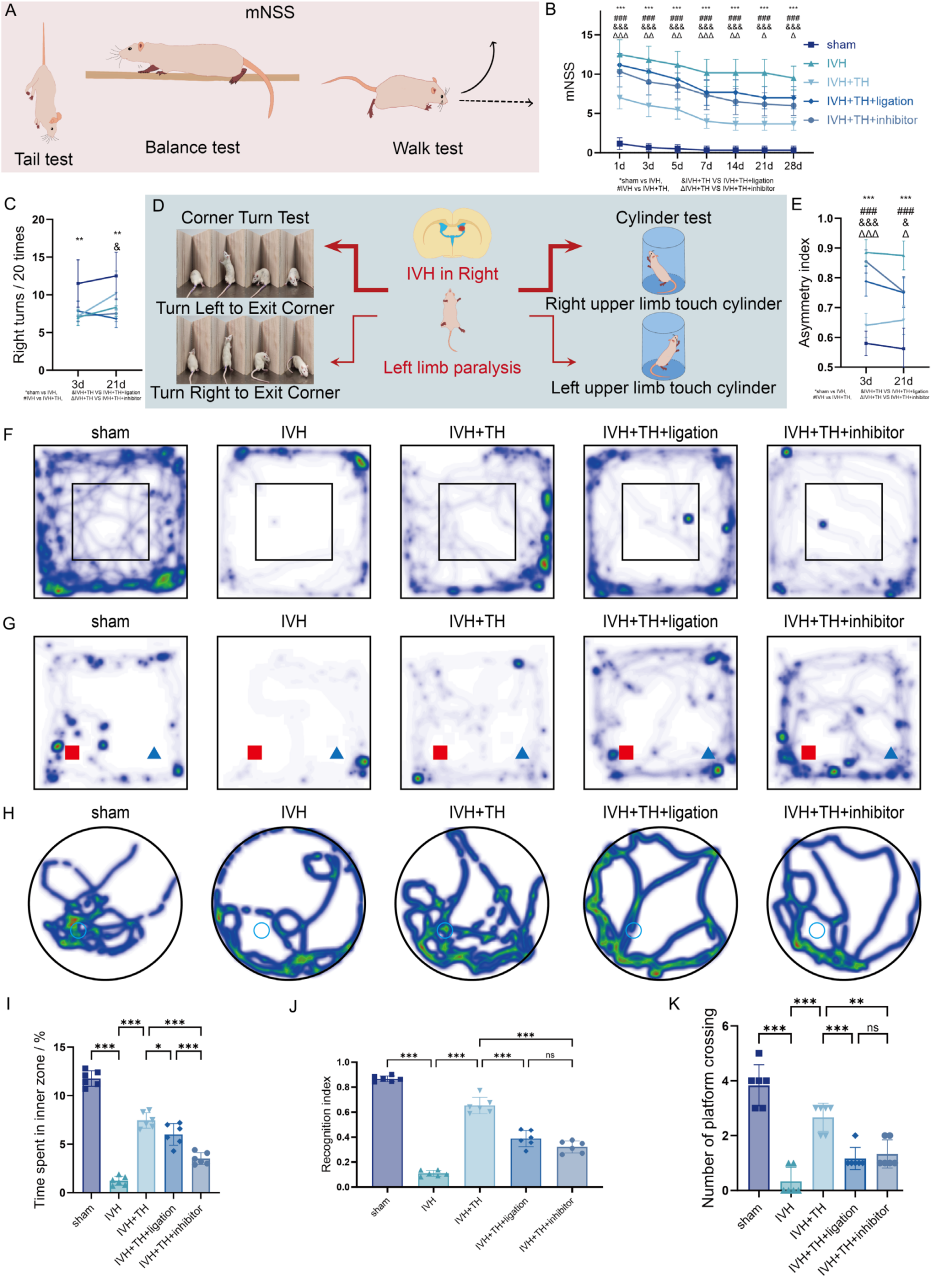
TH alleviates neurological dysfunction post IVH in rats. (A) Schematic diagram of mNSS (part of test items). (B) The mNSS of each group at different time points during timeline in experiment. (C) Right turns/20 times of each group in corner turn test. (D) Schematic diagram of corner turn test and cylinder test. (E) Asymmetry index of each group in cylinder test. (F) Representative density map of each group in OFT. (G) Representative density map of each group in NOR. (H) Representative density map of each group in MWM. (I) Time spent in inner zone of each group in OFT. (J) Recognition index of each group in NOR. (K) Number of platform crossing of each group in MWM. All data are presented as mean ± SD (*n* = 6 per group). Significance levels are denoted as: *p < 0.05, **p < 0.01, ***p < 0.001.

In order to evaluate comprehensive neurological function of rats, mNSS was employed, encompassing evaluations of motor coordination, sensory perception, and postural reflexes (Figure 1A). Elevated mNSS correlates with greater neurological dysfunction in rats. IVH group demonstrated significantly higher scores compared to sham. TH significantly reduced these scores, whereas ligation or inhibitor partially reversed this improvement. Notably, spontaneous neurological recovery observed over time remained incomplete (Figure 1B). Corner turn test and cylinder test are commonly used to detect the asymmetric injury of bilateral limbs of rats, and the number of escaping angles from the right side and asymmetric index of upper limb touching cylinder is used to reflect limb function (Figure 1D). The results exhibited marked ipsilateral bias of rats in IVH group, but it improved after TH. This therapeutic effect will be partially reversed by ligation and inhibitor (Figure 1C,E). OFT is often used to evaluate the anxiety of rats. The more time or distance they spend in the inner zone of the field, the higher the desire of rats to explore unfamiliar environments (Figure S3B). IVH group demonstrated significant thigmotaxis in outer zone or even corners, and they spent more time in outer zone. After TH, the willingness to move to the inner zone increased, but it was reversed after ligation or inhibitor (Figure 1F,I, Figure S3A). To assess short‐term cognition and memory in rats, NOR were used for the study (Figure S3C). The recognition index and discrimination index reflect the preference degree of rats for new objects. Rats in IVH group could not distinguish new objects from old objects well, and this distinguishing ability was restored to some extent after TH, but this ability was partially lost after ligation or inhibitor (Figure 1G,J and Figure S3D,E). MWM is an experimental project to evaluate long‐term learning and memory ability (Figure S3F). The time they spent in the target quadrant and the number of crossing the platform can explain the memory ability of rats. In IVH group, the memory of rats was significantly impaired, and the learning ability was reduced. However, the memory improved after TH, and ligation and inhibitors played destructive effect partially (Figure 1H,K and Figure S3G–I). Based on the above results, TH alleviates neurological dysfunction post IVH in rats.

### 3.2 TH Attenuates Hydrocephalus and Pathology Damage Post IVH in Rats

**FIGURE 2:**
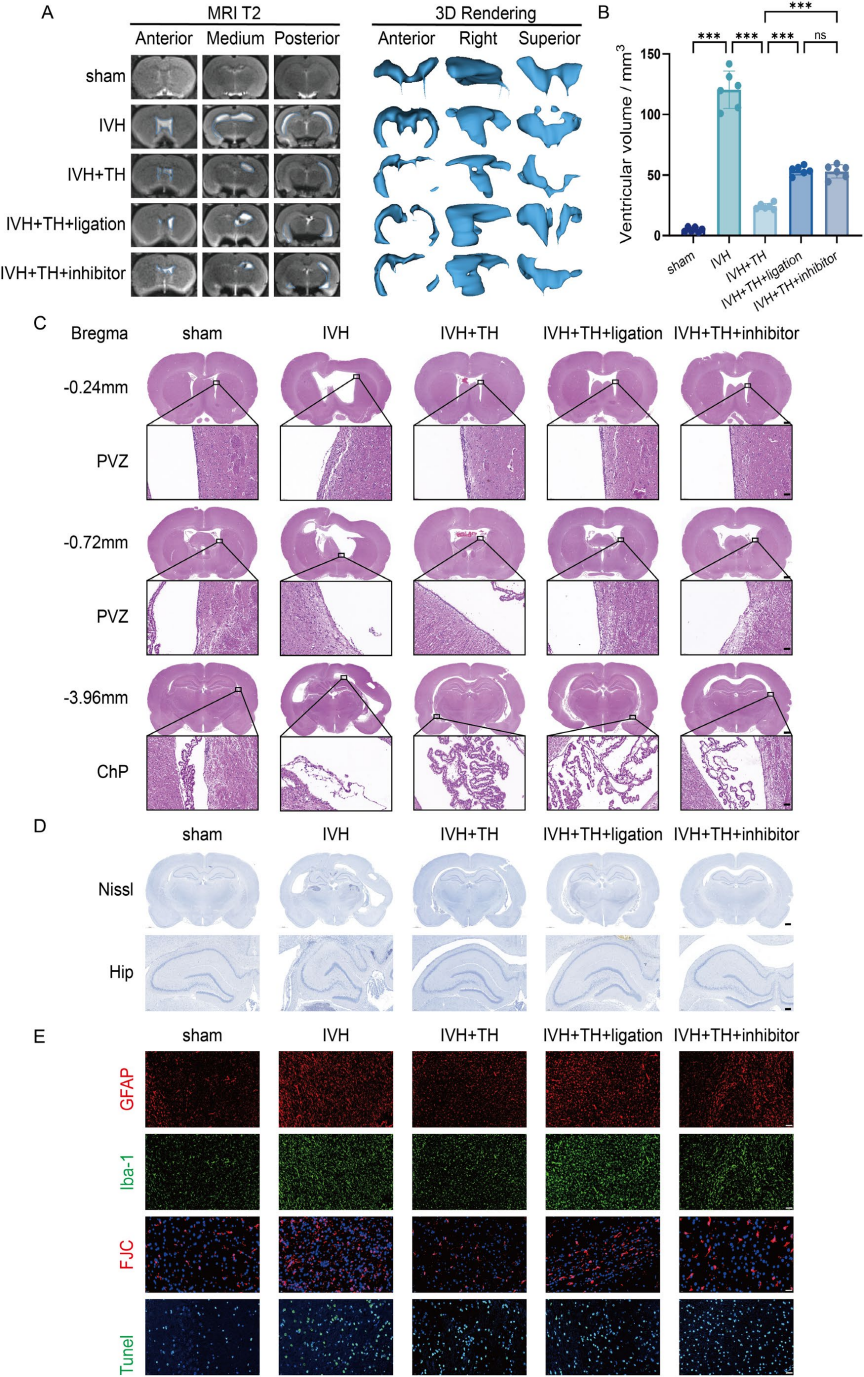
TH attenuates hydrocephalus and pathology damage post IVH in rats. (A) Representative coronal brain plane from anterior to posterior and 3D rendering of each group on MRI T2. (B) Ventricular volume measured by 3D rendering of each group. (C) Representative HE staining in three coronal brain sections at bregma‐0.24 mm, −0.72 mm and −3.96 mm of each group (scale bar = 1000 μm) and zoom for PVZ and ChP (scale bar = 50 μm). (D) Representative Nissl staining in brain (scale bar = 1000 μm) and Hip (scale bar = 250 μm) of each group. (E) Representative IF for GFAP, Iba‐1, FJC and TUNEL of each group (scale bar = 100 μm). All data are presented as mean ± SD (*n* = 6 per group). Significance levels are denoted as: *p < 0.05, **p < 0.01, ***p < 0.001. PVZ, Periventricular zone; ChP, Choroid plexus; Hip, Hippocampus.

Quantitative analysis of ventricular morphology was performed using MRI T2 and 3D rendering, ventricle volumes of rats will be quantified to account for the degree of hydrocephalus (Figure S4A). Rats in the IVH group showed obvious ventricular dilation and CSF accumulation, while after TH, the ventricle volume was reduced and the degree of hydrocephalus was reduced, but hydrocephalus severity was exacerbated by ligation or inhibitor (Figure 2A,B and Figure S4B). After HE staining of brain tissue sections, the pathology damage of brain tissue can be observed, and different parts can be seen at different coronal sectional positions. The IVH group demonstrated ependymal cilia depletion and morphological damage in the periventricular zone (PVZ), while more inflammatory cells can be seen in the choroid plexus (ChP). This suggests damage to the cilia and inflammatory infiltration of ChP. After TH, this injury improved, but ligation or inhibitor aggravated its performance again (Figure 2C). Nissl staining is often used to observe the apoptosis of neurons. The eyes are often focused on the hippocampus (Hip), which can be further subdivided into cornu ammonis 1 (CA1), cornu ammonis 3 (CA3) and dentate gyrus (DG). In the IVH group, the cell morphology was shrunk, the nuclear is fragmentation and lysis, and the number of Nissl bodies was significantly reduced. After TH, the cell morphology remained normal, and the number of Nissl bodies recovered. But ligation or inhibitors reduce this efficacy (Figure 2D, Figure S5A). In order to observe the degree of neuroinflammation in brain tissue, IF staining is often performed with GFAP and Iba‐1. GFAP can represent the reactive proliferation of astrocytes, while Iba‐1 represents the activation of microglia. According to its positive area proportion, it can reflect the severity of neuroinflammation. The IVH group showed more astrocyte proliferation and microglia activation, indicating that the neuroinflammation was more severe. This inflammatory manifestation is alleviated by TH, but the degree of neuroinflammation increases with ligation or inhibitor (Figure 2E and Figure S5B–D). FJC staining and TUNEL staining were used to evaluate degenerative changes and late apoptosis of neurons, respectively. The average fluorescence intensity of FJC and TUNEL in the IVH group was higher, which reflected the severe degeneration and late apoptosis of neurons. In contrast, TH improves this change in neurons, but it is reversed by ligation or inhibitors (Figure 2E, Figure S5B,E,F). After considering the aforementioned results, TH attenuates hydrocephalus and pathology damage post IVH in rats.

### 3.3 TH Protects Physiological Function of Glymphatic System Post IVH in Rats

**FIGURE 3:**
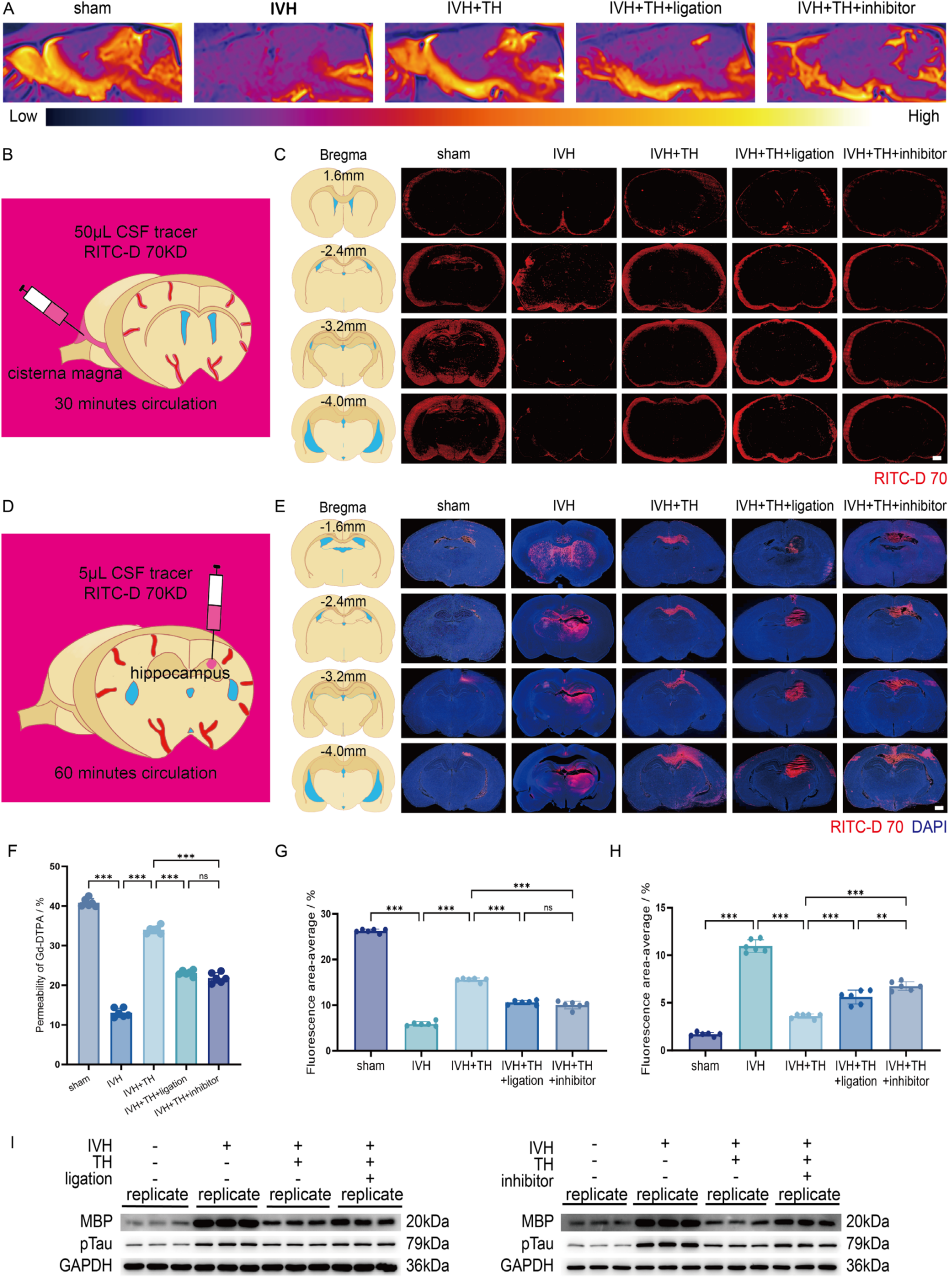
TH protects physiological function of glymphatic system post IVH in rats. (A) Representative sagittal brain plane after injection of Gd‐DTPA processed by pseudo‐color of each group on MRI T1. (B) Schematic diagram of inflow. (C) Representative images for RITC‐D 70 kD accumulation in four coronal brain sections at bregma +1.6 mm, −2.4 mm, −3.2 mm, and −4.0 mm of each group (scale bar = 1000 μm). (D) Schematic diagram of outflow. (E) Representative images for RITC‐D 70 kD accumulation in four coronal brain sections at bregma −1.6 mm, −2.4 mm, −3.2 mm, and −4.0 mm of each group (scale bar = 1000 μm). (F) Quantification for permeability of Gd‐DTPA of each group. (G) Average quantification for fluorescence area covered by RITC‐D 70 kD in four sections per animal of each group. (H) Average quantification for fluorescence area covered by RITC‐D 70 kD in four sections per animal of each group. (I) Western blot scans for MBP, pTau and GAPDH of each group in experiment of dCLNs ligation and in experiment of AMPK inhibitor. All data are presented as mean ± SD (*n* = 6 per group). Significance levels are denoted as: *p < 0.05, **p < 0.01, ***p < 0.001. Gd‐DTPA, Gadopentetate Dimeglumine; RITC‐D 70, Rhodamine B isothiocyanate‐Dextran‐average mol wt‐70,000; DAPI, 4′,6‐diamidino‐2‐phenylindole; MBP, Myelin Basic Protein.

Paramagnetic contrast agent Gd‐DTPA, injected through the cisterna magna, was mixed with the CSF of rats. Observation of the sagittal plane under MRI T1 can evaluate CSF‐brain parenchyma exchange dynamics and thus reflect the permeability of CSF into the brain parenchyma, which can reflect the function of GS. The original image obtained by MRI T1 is represented by gray value. In order to demonstrate distinct spatial distribution patterns of contrast agent infiltration and corroborate quantitative findings of GS dysfunction in IVH pathology, we use ImageJ to process the image pseudo‐color (Figure S6A). Signal intensity quantification revealed that the permeability of Gd‐DTPA into brain parenchyma in IVH group rats was significantly weakened. This permeability is enhanced after TH, but it is inhibited by ligation or inhibitor (Figure 3A,F and Figure S6B). This indicates that the function of GS in IVH rats is dysfunctional, and TH can alleviate this dysfunction, but the alleviation effect is offset by ligation or inhibitor. In addition to the use of contrast agents, the function of the GS can also be evaluated with the aid of fluorescent agents. This experiment is divided into two parts, inflow and outflow. Inflow refers to the injection of fluorescent agent through the cisterna magnum, mixing it with CSF, and observing the distribution of fluorescence in the brain parenchyma after it circulates for a period of time (Figure 3B). The results showed that the fluorescence distribution area in the brain parenchyma of IVH rats was significantly reduced, and the ability of fluorescent agents in CSF to flow into the brain parenchyma was reduced. However, after TH, the fluorescence distribution area increased, the penetration ability of CSF into brain parenchyma increased, and the function of GS was protected. But this protective effect is interfered by ligation or inhibitor (Figure 3C,G and Figure S7A). Correspondingly, in outflow, the fluorescent agent is injected into the brain parenchyma through intrahippocampal injection, and after it circulates for a certain period of time, the fluorescence distribution in the brain is observed to evaluate the clearance ability of the brain parenchyma (Figure 3D). In IVH group, the fluorescent area in brain parenchyma increased significantly, indicating that its ability to remove fluorescent agents from brain parenchyma into CSF decreased, and the function of GS was impaired. After TH, the distribution of fluorescent agents in the brain decreased, indicating that the clearance function of GS was restored. But ligation or inhibitor destroys this recovery effect (Figure 3E,H and Figure S7B). To determine whether TH facilitates the clearance of metabolic waste and cellular debris via the system, we measured the expression levels of myelin basic protein (MBP) and pTau in each group. The results demonstrated that TH significantly enhanced the clearance of both substances, as indicated by their decreased levels in the brain. However, this clearance effect was inhibited when ligation or inhibitor were applied, leading to increased accumulation of MBP and pTau in the brain (Figure 3I and Figure S7C,D). These bidirectional tracer experiments and substances clear experiments confirm TH‐mediated restoration of glymphatic influx/efflux. Taking everything into account, TH protects the physiological function of the glymphatic system post IVH in rats.

### 3.4 TH Maintains Normal Structure of Glymphatic System Post IVH in Rats

**FIGURE 4:**
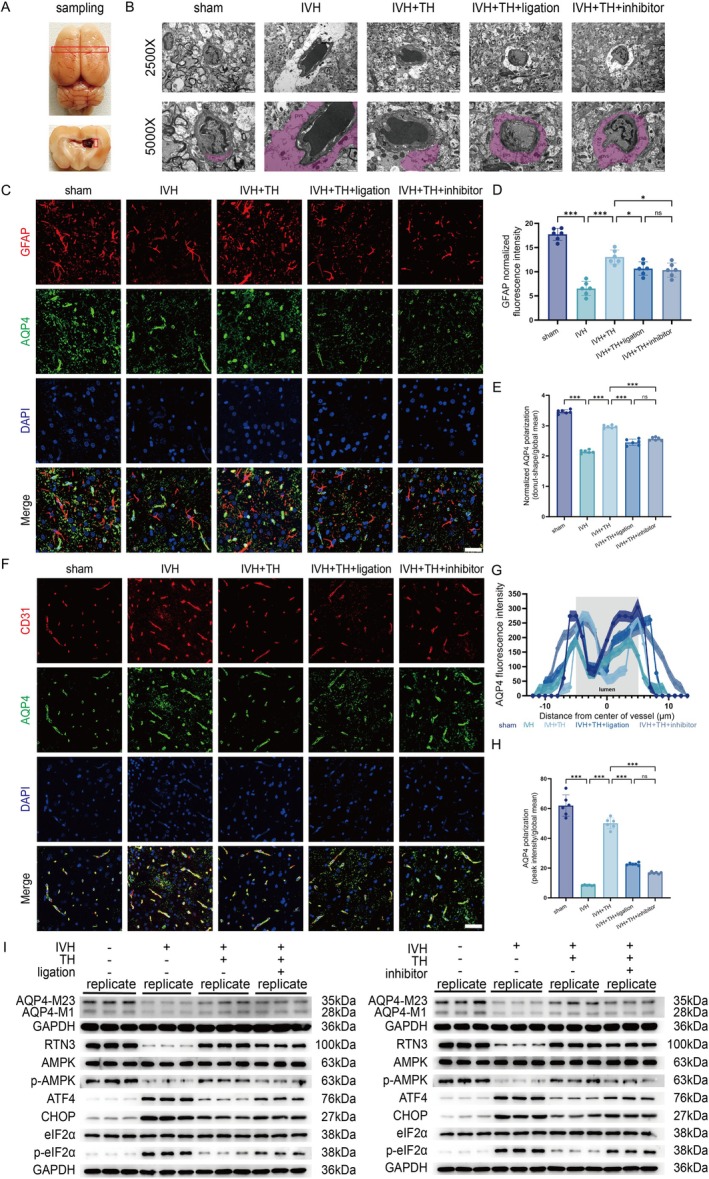
TH maintains normal structure of glymphatic system and regulates RTN3/AMPK/ERS pathway post IVH in rats. (A) Photo of brain in the process of sampling for TEM. (B) Representative images for TEM in 2500X (scale bar = 2 μm) and 5000X (scale bar = 1 μm) of each group. (C) Representative IF for GFAP and AQP4 of each group (scale bar = 50 μm). (D) GFAP normalized fluorescence intensity of each group. (E) Normalized AQP4 polarization (donut‐shape/global mean) of each group. (F) Representative IF for CD31 and AQP4 of each group (scale bar = 50 μm). (G) AQP4 fluorescence intensity of each group. (H) AQP4 polarization (peak intensity/global mean) of each group. (I) Western blot scans for AQP4‐M23, AQP4‐M1, RTN3, AMPK, p‐AMPK, ATF4, CHOP, eIF2α, p‐eIF2α and GAPDH of each group in experiment of dCLNs ligation and in experiment of AMPK inhibitor. All data are presented as mean ± SD (*n* = 6/3 per group). Significance levels are denoted as: *p < 0.05, **p < 0.01, ***p < 0.001. NVU, Neurovascular unit; a, Astrocyte; v, Vessel; ER, Endoplasmic reticulum; PVS, Perivascular space; RTN3, Reticulon 3; AMPK, Adenosine 5′‐monophosphate (AMP)‐activated protein kinase; ATF4, Activating transcription factor 4; CHOP, C/EBP homologous protein; eIF2α, Eukaryotic initiation factor 2α; GAPDH, Glyceraldehyde‐3‐phosphate dehydrogenase.

In TEM, we observed the ultrastructure of NVU in GS from the cellular level. After obtaining tissue of the PVZ (Figure 4A), the results of electron microscopy showed that the overall structure of the micro vessel was abnormal, the structure of the vascular intima was loose, the PVS was extremely dilated in tissue, the end foot of the astrocyte and the vascular basement membrane were obviously loose and edematous, and the endoplasmic reticulum was obviously dilated in tissue. The structural integrity of this NVU is also protected after TH, but it is destroyed by ligation or inhibitors (Figure 4B). In addition to evaluating the structure of GS at the ultrastructure, we employed IF staining to observe it at the molecular level. Astrocytes were calibrated by GFAP and vascular endothelial cells were calibrated by CD31 to determine NVU. On this basis, the fluorescence expression of AQP4 was detected to evaluate the polarization distribution of AQP4 in NVU, and then described the structure of GS. From the results of IF staining, combined with the measurement and analysis of AQP4 polarization described in the previous method, the results delineated that AQP4, which was originally polarized to play the role of aquaporin, showed a loss of polarity after IVH and a depolarization distribution phenomenon, which destroyed the integrity of NVU and led to damage to the GS structure. However, after TH, the polarization distribution of AQP4 recovered and the structure of GS was protected. This protective effect was attenuated by ligation or inhibitors (Figure 4C–H). In addition, the polarization of AQP4 can also be evaluated in WB by ratio analysis for different isoforms of AQP4, which yielded the same results as in IF (Figure 4I and Figure S7E,F). Given the above points, TH maintains the normal structure of the glymphatic system post IVH in rats.

### 3.5 TH Upregulates RTN3, Facilitates Phosphorylation of AMPK and Suppresses ERS Post IVH in Rats

We obtained an ICH database that closely mimics IVH conditions. Analysis revealed that the expression of the *Prkaa2* gene (encoding AMPKα2) was downregulated following ICH (Figure S8A). We then consulted a TH database, which, although based on a TBI model, offers relevant insights into TH effects. The analysis showed that expression of the *Prkab1* gene (encoding AMPKβ1) was downregulated after TBI but upregulated following TH. In addition, the genes *Prkab2* (AMPKβ2) and *Prkaa1* (AMPKα1) were both upregulated when TH was applied after TBI. These findings provide preliminary evidence supporting a role for AMPK in the mechanism of TH for IVH (Figure S8B–D). And then, we used WB to detect the expression of related molecular proteins (Figure 4I and Figure S7G–K). After TH, the expression of RTN3 increased, the phosphorylation level of AMPK signaling pathway increased, and the contents of proteins related to ERS such as ATF4, CHOP, and eIF2α were inhibited. Either ligation or inhibitor can reverse the changes in molecular protein expression caused by TH. In other words, ligation or inhibitor destroys the efficacy of TH. Synthesizing the key findings, TH upregulates RTN3, facilitates phosphorylation of AMPK, and suppresses ERS post IVH in rats.

### 3.6 TH Improves Function and Structure of Meningeal Lymphatic System Post in Rats

**FIGURE 5:**
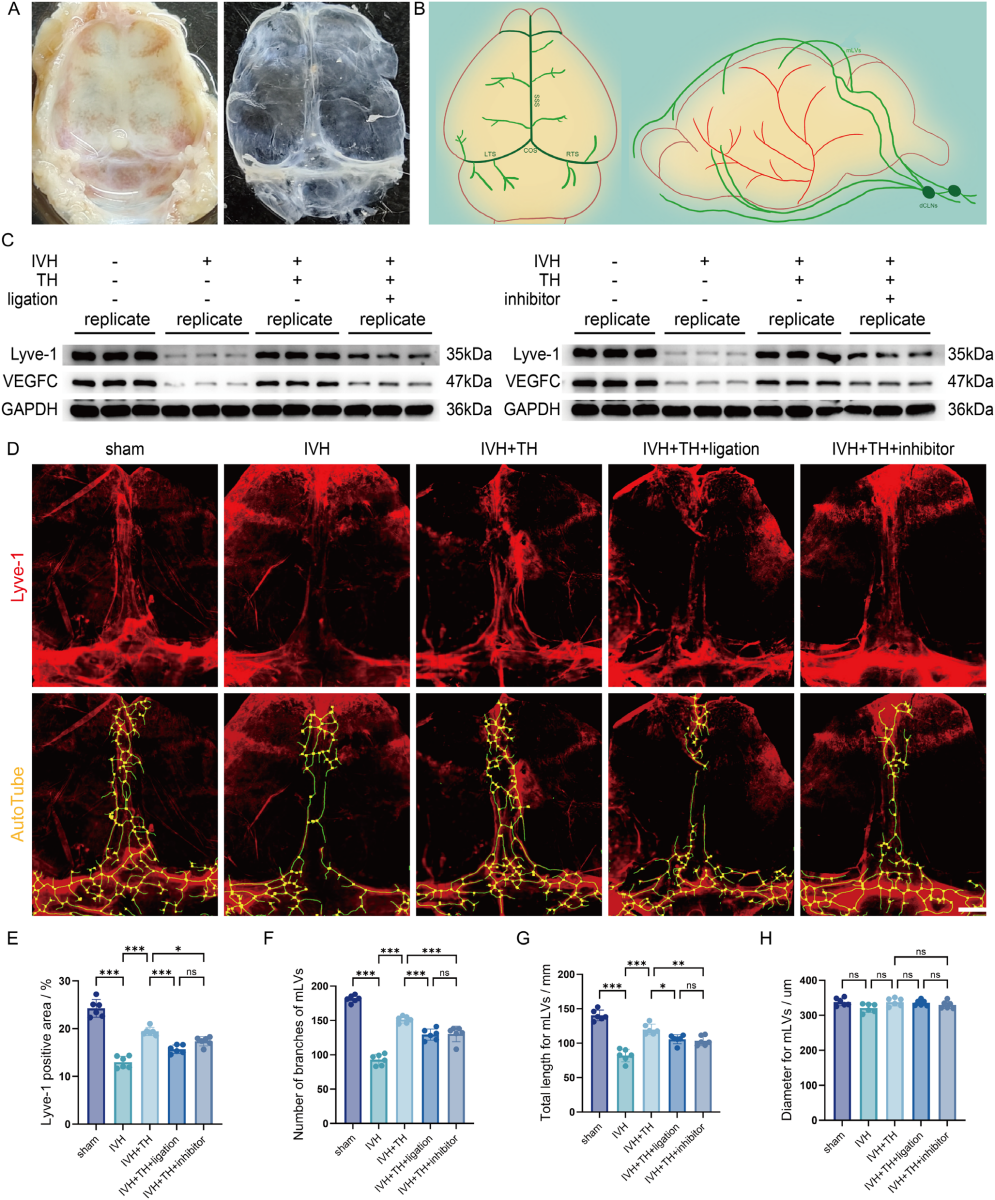
TH improves function and structure of meningeal lymphatic system post in rats. (A) Photo of parietal bone and meninge in the process of sampling. (B) Schematic diagram of mLVs and dCLNs. (C) Western blot scans for Lyve‐1, VEGFC, and GAPDH of each group in experiment of dCLNs ligation and in experiment of AMPK inhibitor. (D) Representative images for meninges stained by Lyve‐1 and branches of mLVs acquired by AutoTube of each group (scale bar = 2000 μm). (E) Quantification for Lyve‐1 positive area of each group. (F) Number of branches of mLVs of each group. (G) Total length for mLVs of each group. (H) Diameter for mLVs of each group. All data are presented as mean ± SD (*n* = 6/3 per group). Significance levels are denoted as: *p < 0.05, **p < 0.01, ***p < 0.001. mLVs, Meningeal lymphatic vessels; SSS, Superior sagittal sinus; COS, Confluence of sinuses; LTS, Left transverse sinus; RTS, Right transverse sinus; Lyve‐1, Lymphatic vessel endothelial hyaluronan receptor 1; VEGFC, Vascular endothelial growth factor C.

As the final output structure of GS, mLVs can be referred to as constituent components of GS in a broad sense (Figure 5A,B). To evaluate its function, the changes of protein expression levels in meninges were detected by WB, and the semi‐quantitative analysis of meningeal lymphatic vessels was performed by Lyve‐1 and VEGFC. The results indicated that the function of the meningeal lymphatic system is damaged after IVH, which also means that its drainage and exclusion function decreases. However, after TH, expression of Lyve‐1 and VEGFC was increased, indicating that TH protected the function of the meningeal lymphatic. This protective effect is blocked by ligation or inhibitor (Figure 5C and Figure S7L–M). Lyve‐1 was used to label mLVs, and its morphological structure can be observed in IF staining, and the changes of branches, length, and diameter can be analyzed by AutoTube (Figure 5D). The results showed that in the IVH group, the overall coverage of Lyve‐1's on meninges decreased, the number of branches of mLVs decreased, and the total length of mLVs decreased. This indicates that the meningeal lymphatic structure is damaged after IVH, which also means that its drainage and exclusion function decreases. However, after TH, the coverage of Lyve‐1 increased, the number of branches of mLVs increased, and the total length also prolonged, indicating that TH protected the structure of the meningeal lymphatic. This protective effect is blocked by ligation or inhibitor (Figure 5E–G). However, there seemed to be no obvious difference in the diameter of mLVs between the groups of rats (Figure 5H). Does this indicate that the damaging effect of IVH and the protective effect of TH did not affect the tube diameter alone? We further investigated the role of mLVs in TH for hydrocephalus post IVH. After injecting visudyne into the cisterna magna, we ablated the mLVs via laser irradiation and performed MRI scans. T2‐weighted imaging revealed that visudyne reversed the beneficial effect of TH in alleviating ventricular dilation (Figure S9A,B). Meanwhile, T1‐weighted imaging showed reduced penetration of contrast agent, suggesting impaired glymphatic function (Figure S9C,D). Behavioral assessments, including the OFT and MWM, further indicated that visudyne treatment exacerbated neurological impairment in rats (Figure S9E–H). This result needs to be further studied. Upon reviewing the data, TH remedy's function and structure of the meningeal lymphatic system post in rats.

### 3.7 TH Promotes Drainage of Deep Cervical Lymphatic System Post IVH in Rats

**FIGURE 6:**
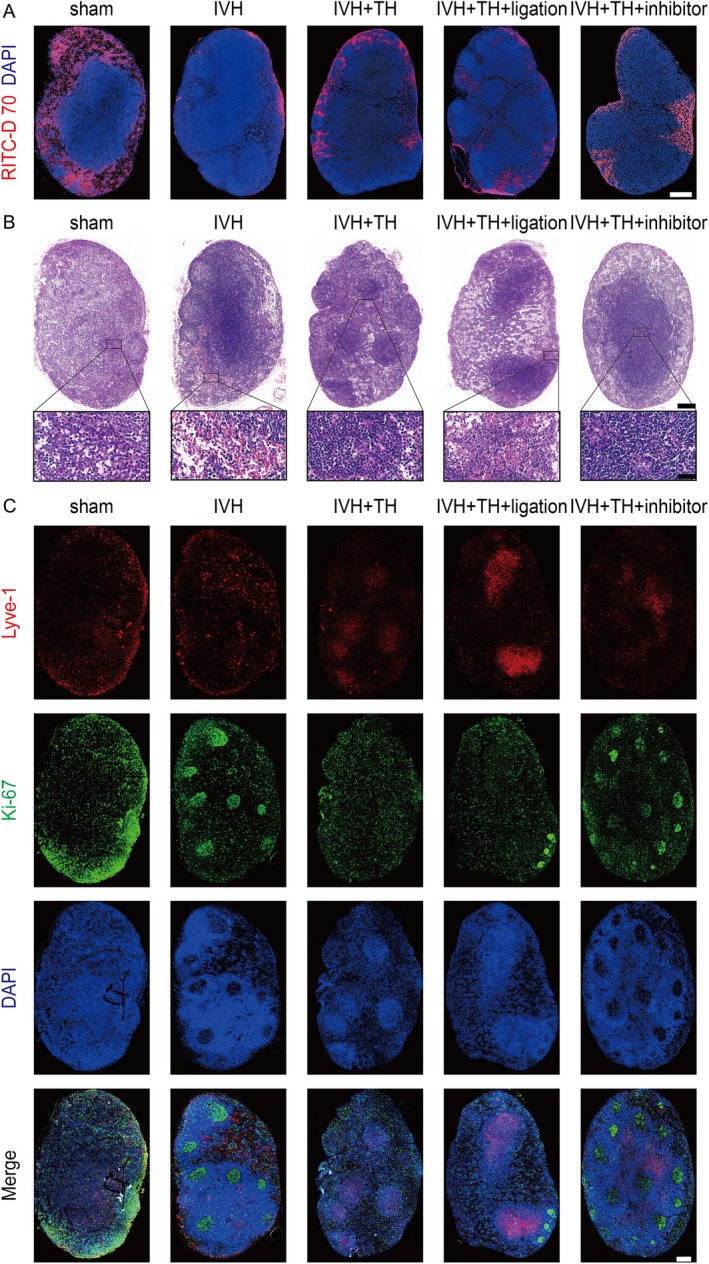
TH promotes drainage of deep cervical lymphatic system post IVH in rats. (A) Representative images for RITC‐D 70kD accumulation in dCLNs of each group (scale bar = 500 μm). (B) Representative HE staining in dCLNs of each group (scale bar = 200 μm and 10 μm in zoom). (C) Representative IF for Lyve‐1 and Ki‐67 in dCLNs of each group (scale bar = 200 μm).

In order to evaluate the role of drainage function of dCLNs in protecting the GS of TH, we simultaneously collected dCLNs and detected the distribution of fluorescence in them in the influx experiment mentioned earlier (Figure 3B). The results showed that the fluorescence distribution in dCLNs in IVH group was less, indicating that the drainage of fluorescent agent to dCLNs was compromised, while the fluorescence distribution range in dCLNs increased after TH, indicating that the drainage function of dCLNs was improved. But the effect of this elevation was reduced again by ligation or inhibitor (Figure 6A and Figure S7N). Subsequently, we made sections of dCLNs and performed HE staining to observe the tissue damage. The lymph node cells of IVH rats were swollen, disordered, inflammatory cell infiltration increased, and even a small number of red blood cells were mixed. Fortunately, this pathological injury improved after TH, but ligation or inhibitor aggravated this injury (Figure 6B). After that, we used IF staining to evaluate the proliferation of lymphatic vessels in dCLNs, and Pearson correlation coefficient to quantitatively analyze the colocalization between Lyve‐1 and Ki‐67. The results showed that in the dCLNs of IVH rats, the colocalization coefficient of Lyve‐1 and Ki‐67 was significantly reduced, indicating that the proliferation of lymphatic vessels was hindered, the structure was damaged, and then the drainage function was impaired. After TH, its colocalization improved, the lymphatic structure was protected, and its drainage function was restored. While ligation or inhibitor inhibited the protective effect of TH. (Figure 6C and Figure S7O). In light of the findings, TH promotes drainage of deep cervical lymphatic system post IVH in rats.

### 3.8 TH Protects Astrocytes and Lymphatic Endothelial Cells Directly by Inhibiting ERS

**FIGURE 7:**
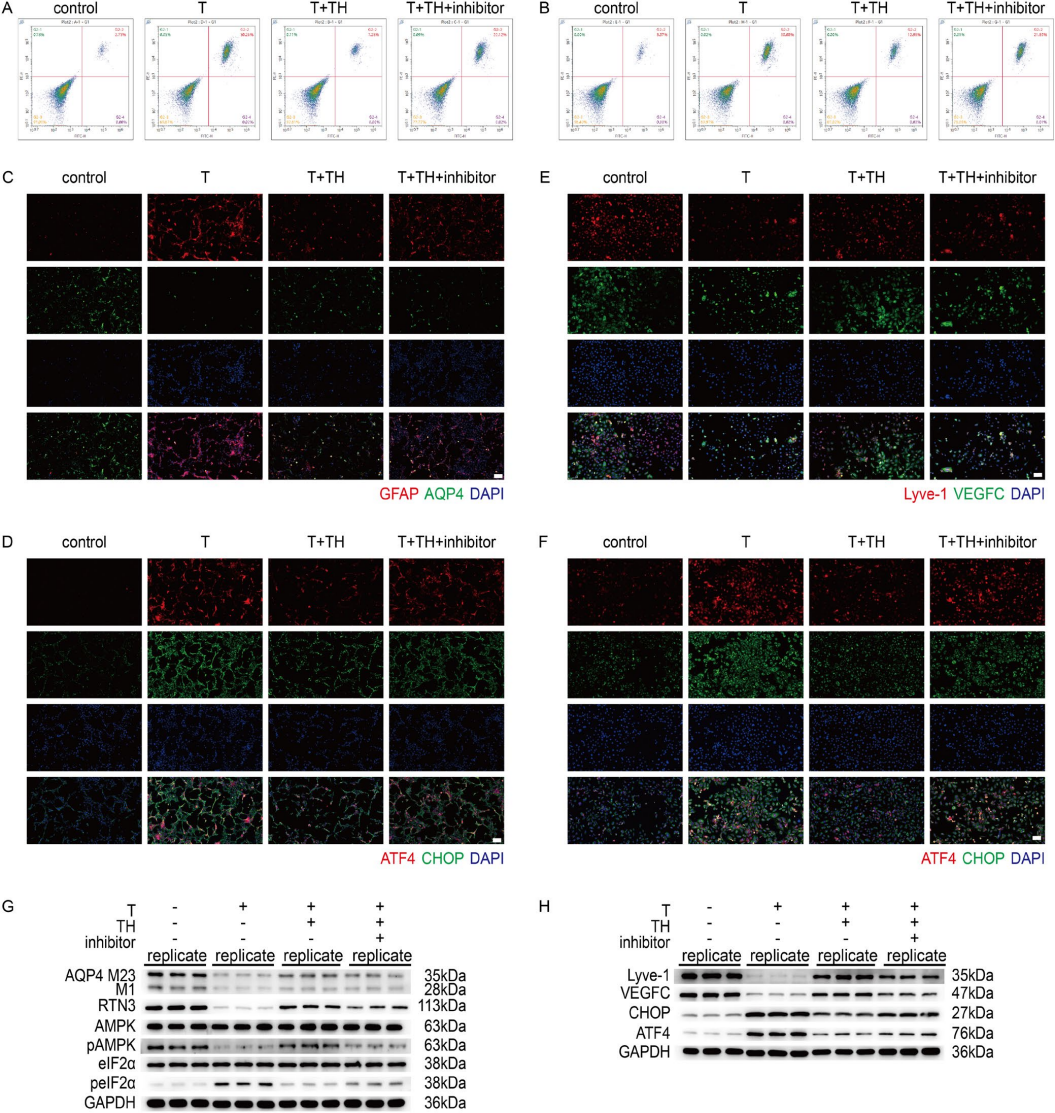
Exploration of mechanisms at the cellular level post IVH. (A) Representative images for apoptosis in flow cytometry in astrocytes of each group. (B) Representative images for apoptosis in flow cytometry in lymphatic endothelial cells of each group. (C) Representative IF for GFAP and AQP4 in astrocytes of each group (scale bar = 50 μm). (D) Representative IF for ATF4 and CHOP in astrocytes of each group (scale bar = 50 μm). (E) Representative IF for Lyve‐1 and VEGFC in lymphatic endothelial cells of each group (scale bar = 50 μm). (F) Representative IF for ATF4 and CHOP in lymphatic endothelial cells of each group (scale bar = 50 μm). (G) Western blot scans for AQP4‐M23, AQP4‐M1, RTN3, AMPK, p‐AMPK, eIF2α, p‐eIF2α and GAPDH in astrocytes of each group. (H) Western blot scans for Lyve‐1, VEGFC, CHOP, ATF4 and GAPDH in lymphatic endothelial cells of each group.

In animal models, we previously demonstrated the protective role of TH against hydrocephalus post IVH, which was mediated through the facilitation of the glymphatic‐meningeal lymphatic‐deep cervical lymphatic system. To further investigate the direct cellular effects of TH, we conducted in vitro experiments using rat astrocytes to model the glymphatic system and mouse lymph node endothelial cells to represent the meningeal and deep cervical lymphatic systems. First, flow cytometry was performed to assess apoptosis in both cell types. The results indicated that TH treatment reduced apoptosis, while this protective effect was reversed by AMPK inhibitors (Figure 7A,B, Figures S10A,B and S11A,B). Subsequently, IF was employed to examine the expression of GFAP and AQP4 in astrocytes, and Lyve‐1 and VEGFC in lymphatic endothelial cells. These markers reflect cellular integrity and functional states, and the findings revealed that TH treatment alleviated injury and supported cellular function (Figure 7C,E, Figure S10C,D,G,H). In addition, ERS markers in both cell types were suppressed by TH (Figure 7D,F, Figure S10E,F,I–J). Finally, protein‐level analysis confirmed that TH upregulates RTN3 expression in astrocytes, promotes AMPK phosphorylation, and inhibits ERS (Figure 7G, Figure S10K–O). Similarly, in lymphatic endothelial cells, TH also attenuated ERS (Figure 7H, Figure S10P–S). Collectively, these results demonstrate that TH directly suppresses astrocyte activation and preserves the function of lymphatic endothelial cells.

## 4 Discussion

Hydrocephalus is a disease in which CSF accumulates excessively in the ventricular system due to the imbalance of CSF circulation homeostasis [37, 38]. Neurosurgeons mostly use surgeries for the treatment of hydrocephalus post IVH [39]. However, surgical intervention is always an invasive operation, whether it is the damage to the patient's body caused by the operation itself, or the routine postoperative complications such as bleeding, infection and recurrence, or even the special occurrence of shunt rupture, shunt blockage, shunt‐dependent syndrome [40], slit ventricle syndrome [41], Sylvian aqueduct syndrome [42] and so on. All these have caused great inconvenience to patients, which not only affects their life and health, but also affects their work and economy. Therefore, it is of high practical clinical value to study the pathogenesis of hydrocephalus post IVH and explore the application of novel therapeutic strategy.

In this study, we have found that in hydrocephalus post IVH, the function of the glymphatic‐meningeal lymphatic‐deep cervical lymphatic system was impaired, which was manifested by decreased permeability of paramagnetic contrast agent Gd‐DTPA from CSF to brain parenchyma on TMRI T1, decreased permeability of fluorescent agent RITC‐D 70 from CSF to brain parenchyma, and decreased clearance of fluorescent agent RITC‐D 70 from brain parenchyma to CSF, which was then deposited in the brain parenchyma. There was also a decrease in the drainage of the fluorescent agent RITC‐D 70 into the dCLNs. In the observation of the structural damage, it was found that at the molecular level, the key core component, AQP4, was depolarized, lost its normal subcellular localization, and shifted to the intracellular region from the astrocyte end‐foot membrane. At the ultrastructural level, swelling of astrocytes, enlargement of the PVS, and swelling and deformation of the ER in astrocytes were observed. Subsequently, with the aid of IF staining, damage to the mLVs was observed, as evidenced by a reduction in the number of branches and the total length of the ducts. Structural damage and dysfunction of the dCLNs were also observed. It was found that in hydrocephalus post IVH, the disruption of the glymphatic‐meningeal lymphatic‐deep cervical lymphatic system could be a novel target for treatment.

In addition to our research, dysfunction of the glymphatic‐meningeal lymphatic‐deep cervical lymphatic system is closely associated with a variety of neurological disorders, which have embodied its importance in neurological disorders. Studies have shown that the clearance efficiency of the GS decreases significantly during aging, which may be one of the important mechanisms for amyloid β deposition in neurodegenerative diseases such as Alzheimer's disease [43]. With more advanced detection technology and the support of near‐infrared fluorescence imaging technology, researchers found that TH can increase the inflow of GS by reducing the flow rate of CSF [44]. In models of TBI, impaired clearance of the GS leads to toxic protein accumulation and increased neuroinflammation [45]. In a rat model of cerebral ischemia, acute inhibition of AQP4 using TGN‐020 attenuated early cerebral edema, and also inhibited peri‐infarct astrocyte responses and depolarization of AQP4 in the subacute phase, thereby promoting neurological recovery [46, 47]. Marie‐Gabrielle Duperron proposed that enlarged PVS located in the basal ganglia and hippocampus is significantly associated with an increased risk of intracerebral hemorrhage [48]. Furthermore, Wenchao Chen demonstrated that mifepristone upregulated AQP4 expression to promote its perivascular polarization profile to improve GS function to promote post‐ICH hematoma clearance [49]. Dongyu Li used 4‐day‐old male rat pups to mimic preterm infants and found that non‐invasive transcranial near‐infrared light could improve GS clearance of drainage and lead to the recovery of IVH, providing a clinical therapeutic technique that may be used for IVH in neonates [50]. With further research, TH has a mechanism of neuroprotective function, which is considered to be related to GS. However, some studies have reported contrasting findings, suggesting that hypothermia may impair glymphatic drainage after TBI. To reconcile these conflicting conclusions, we propose several potential explanations. First, the disease models differ fundamentally. TBI is primarily induced by external trauma, which often leads to significant cerebral edema, while IVH results from vascular rupture and typically presents with hydrocephalus under relatively milder stimuli. These distinct pathogenic and pathophysiological mechanisms may underlie the differential effects of hypothermia. Additionally, the timing of assessment varies across studies. Evaluations conducted in the hyperacute phase after TBI (within 1 day) may capture initial suppression of glymphatic function. In contrast, our observations during the acute phase following IVH (at 3 days) indicate a promotive effect, possibly reflecting temporal shifts in glymphatic activity as the brain progresses through different stages of damage and repair [51, 52]. These studies support our view that GS plays a crucial role in the role of TH for hydrocephalus post IVH.

Since the discovery of mLVs in 2015 [17], there is growing evidence that the meningeal lymphatic system plays a key role in regulating immune responses and inflammation in the central nervous system. Studies have shown that mLVs act as a bridge between the central nervous system and the peripheral lymphatic system and are involved in the drainage of CSF and interstitial fluids, which in turn affects neuroinflammation, immune responses, and waste removal. In Alzheimer's disease, dysfunction of mLVs may lead to reduced clearance of amyloid‐β, thereby exacerbating the pathologic process [53]. In addition, mLVs play a key role in the inflammatory response after TBI and stroke, influencing disease recovery and prognosis by regulating immune cell migration and inflammatory factor release [45]. Qiang Zhang found that extracellular traps of neutrophils activate the CX3CR1 molecule, which induced endothelial cell injury and thrombosis in meningeal lymphatics, disrupting the drainage function of the meningeal lymphatic system and exacerbating brain injury with secondary chronic hydrocephalus after IVH [54]. Therefore, the meningeal lymphatic system plays the communication role as an intermediate bridge in connecting GS and deep cervical lymphatic system as a whole.

Researchers found the role of deep cervical lymphatic system in the formation of hydrocephalus during the chronic phase of IVH [55]. In diseases such as meningitis and multiple sclerosis, dCLNs are involved in the initiation and regulation of systemic immune responses by receiving antigens and immune cells from the central nervous system [56]. It has been found that the immune activation status of dCLNs can influence the severity and duration of neuroinflammation. For example, in a model of experimental autoimmune encephalomyelitis, resection or functional suppression of dCLNs significantly attenuated the severity of the disease, suggesting their critical role in neuroimmune diseases [57]. In a mouse model of Alzheimer's disease, ablation of lymphatic drainage using verteporfin photo‐ablation or lymphatic vessel ligation resulted in amyloid β deposition in the meninges and hippocampus [58]. Furthermore, recent studies have shown that VEGFC‐driven lymphatic drainage of the meninges is critical for modulating depressive behavior and generating effective immune responses against brain tumors [59]. Thus, the deep cervical lymphatic system, as the terminal link, undertakes the final task of clearance and drainage.

In an in vivo experimental animal model, we observed the disruption of the GS in rats with hydrocephalus post IVH, and then we went from the macroscopic to the microscopic to explore the alterations in protein expression in hydrocephalus post IVH at the molecular level. We found that the expression of RTN3 was decreased, the phosphorylation level of AMPK, a key molecule of the AMPK signaling pathway, was reduced, and ERS‐related proteins including ATF4, CHOP, and eIF2α were all overexpressed. In addition, the expression of AQP4, which reflects the structure and function of GS, showed depolarization, and the expression of Lyve‐1 and VEGFC, which reflects the structure and function of the mLVs, was reduced as well. However, the precise mechanism through which TH acts on cellular components remains unclear. To address this, we employed astrocytes to model the glymphatic system and lymph node endothelial cells to represent the meningeal and deep cervical lymphatic systems. Our results demonstrated that TH directly reduces the apoptosis rate induced by thrombin in both cell types and suppresses ERS. Furthermore, TH attenuated inflammatory activation in astrocytes and increased AQP4 expression, while in lymphatic endothelial cells, it up‐regulated the expression of Lyve‐1 and VEGFC, thereby helping to maintain endothelial functional stability. It should be noted, however, that our findings are limited to the direct effects of TH on cellular structure and function. We acknowledge that potential indirect effects mediated by other cell types were not explored in this study. It followed that in hydrocephalus post IVH, the expression change of the RTN3/AMPK/ERS pathway may be a new molecular mechanism concomitant with neurofunctional recovery.

It is not clear what molecular mechanism is used to realize the neuroprotective effect of TH at present. Previous study has found TH restored the neuronal network cultured in vitro in the late stage of hypoxia [60]. In the oxygen–glucose deprivation model, it was observed that TH can inhibit the massive production of hydrogen peroxide and nitric oxide, which significantly reduces the level of oxidative stress and protects the activity of neurons [61]. Further research found that TH combined with intraarterial perfusion of magnesium ion solution can regulate intracellular calcium ion homeostasis in NVU, thereby protecting the integrity of the blood–brain barrier and sufficient cerebral blood flow perfusion, and ultimately achieving the effect of improving neurological deficits [62]. The deficiency of RTN3 located in the endoplasmic reticulum increases the activity of BACE1 and promotes amyloid deposition in a mouse model of Alzheimer's disease; RTN3 thus showed its neuroprotective effect [63]. 147 plays a role in inhibiting ERS in neuronal through AMPK/SREBP‐1 signaling pathway, thus protecting against TBI [64]. Endoplasmic reticulum stress was found in brain tissue in a mouse model of intraventricular hemorrhage. Intraperitoneal injection of the selective GPR120 agonist TUG‐891 inhibited endoplasmic reticulum stress and protected neurons [29]. It can be seen that each component of RTN3/AMPK/ERS signaling pathway is not only related to neuroprotective effect, but also combined with cold stress response, and there is a possibility of interaction among them. Therefore, this study selected RTN3/AMPK/ERS pathway as a possible target of the molecular mechanism of neuroprotective effect of TH, and verified that activating the expression of RTN3 and AMPK or inhibiting the ERS may be a contributory role in neuroprotective function.

Of course, due to the inherent realistic conditions, this study still has certain limitations. First, all results are based on in vivo experiments in animal models, and there is a lack of cross‐validation of in vitro experiments on cellular models. This is because single cell culture cannot well simulate the disease model of hydrocephalus post IVH, nor can it establish the glymphatic‐meningeal lymphatic‐deep cervical lymphatic system which has multicellular components. Of course, this also provides ideas for subsequent in‐depth research. By establishing a multicellular co‐culture system to simulate the microstructure of NVU or developing organoid models, this is also the next work goal of the research team in this paper. Secondly, this study focuses on the discovery and description of phenomena, and the verification and exploration of the subsequent molecular mechanism are not deeply perfect enough. We did not perform knockout intervention for RTN3, so this only reflects that the protection of TH depends on changes in RTN3. Of course, the experimental evidence at the phenomenal level supports the next in‐depth study of the interaction mechanism between RTN3/AMPK/ERS, thus providing a solid theoretical basis for the further adoption of targeted drug intervention. Finally, this study lacks the validation of clinical data, and only provides theoretical support at the animal experimental level, but no further research on humans. In order to truly achieve clinical transformation, the research team of this paper will first use retrospective research to conduct preliminary exploration based on the theoretical basis of animal experiments of this study, combined with the actual clinical work, without affecting patients, and then conduct randomized controlled trials based on the results of retrospective research to provide higher‐level research evidence.

## 5 Conclusion

To conclude, the data in this study demonstrated that TH alleviates hydrocephalus and neurological dysfunction post IVH. It is achieved by protecting the function and structure of the glymphatic‐meningeal lymphatic‐deep cervical lymphatic system to clear hematoma and maintain CSF homeostasis. Its molecular mechanism may be associated with the cold stress response to activate the expression of RTN3, and then enhance the phosphorylation of AMPK to inhibit ERS. In this way, this paper explores the alleviating effect and neuroprotective function of TH, which is the newly proposed but still controversial, on hydrocephalus post IVH, and explains the general and molecular mechanisms from the macro and micro perspectives, so that we can provide a reliable theoretical basis for the application of TH as a novel therapeutic strategy in the clinical treatment of hydrocephalus post IVH.

## Author Contributions

Y.Y., G.X., L.Z., and Q.Z. were responsible for the conception design. Y.Y., Q.O., Y.S., J.W., and K.W. performed the experiments. Y.Y. and Y.S. wrote the initial manuscript. Y.Y., J.W., and K.W. prepared the figures. A.L., Y.Y., Z.L., J.H., Z.Z., L.W., Y.Y., and C.W. analyzed and interpreted the data. G.X., L.Z., Q.Z., and Z.S. conducted a critical review of the paper. G.X. provided financial support. All authors read and approved the final manuscript.

## Funding

This work was supported by grants from National Natural Science Foundation of China (No. 82371362 and No. 82171347) and the Students Innovations in Central South University of China (S202510533551, S202510533455, S202510533552).

## Ethics Statement

All practices on animals were performed in line with the National Institutes of Health Guide for the Care and Use of Laboratory Animals. The experiments were conducted in accordance with the approved guidelines and received approval from the Experimental Animal Welfare and Ethics Review Committee at Xiangya hospital of Central South University (202503038).

## Conflicts of Interest

The authors declare no conflicts of interest.

## Supporting information


**Table S1:** Numbers of animal used in experiment.
**Table S2:** Antibodies used in immunofluorescence.
**Table S3:** Antibodies used in western blot.
**Figure S1:** Illustration of the experimental design and groups.
**Figure S2:** Establishment of animal model and key experimental steps.
**Figure S3:** Behavioral test.
**Figure S4:** MRI T2 sequence.
**Figure S5:** Brain section.
**Figure S6:** MRI T1 sequence.
**Figure S7:** Statistical analysis.
**Figure S8:** Propose possible key roles of AMPK based on existing databases.
**Figure S9:** Hydrocephalus and neurological dysfunction in artificial meningeal destruction group.
**Figure S10:** Statistical analysis.
**Figure S11:** Primary images for flow cytometry.

## Data Availability

The data that support the findings of this study are available from the corresponding author upon reasonable request.
