# Supplementary material for: Therapeutic Hypothermia Alleviates Hydrocephalus and Neurological Dysfunction Post Intraventricular Hemorrhage by Enhancing Drainage of Glymphatic‐Meningeal Lymphatic‐Deep Cervical Lymphatic System
Source: CNS Neurosci Ther. 2026 Jan 24;32(1):e70740. doi: 10.1002/cns.70740 (PMC12831171; doi:10.1002/cns.70740)
Supplement: Supplementary file 1 — Table S1: Numbers of animal used in experiment. Table S2: Antibodies used in immunofluorescence. Table S3: Antibodies used in western blot. Figure S1: Illustration of the experimental design and groups. Figure S2: Establishment of animal model and key experimental steps. Figure S3: Behavioral test. Figure S4: MRI T2 sequence. Figure S5: Brain section. Figure S6: MRI T1 sequence. Figure S7: Statistical analysis. Figure S8: Propose possible key roles of AMPK based on existing databases. Figure S9: Hydrocephalus and neurological dysfunction in artificial meningeal destruction group. Figure S10: Statistical analysis. Figure S11: Primary images for flow cytometry. [file CNS-32-e70740-s001.docx]

**Supplementary File**

**Appendix S1:**

**Intraventricular hemorrhage model:** Briefly, the rats were anesthetized with 10% pentobarbital (40mg/kg) and secured on a stereotaxic instrument (RWD, China). Autologous blood (200 μL) was injected in right lateral ventricle with a microsyringe and microinfusion pump at a speed of 20 μL/min (coordinates: bregma, posterior 0.72 mm; lateral 1.60 mm; depth 4.00 mm). The device was left in place for 5 minutes before we closed the cranial foramen and scalp incision.

**Therapeutic hypothermia:** After the completion of surgery, rats were put on the heating pad immediately with rectal temperature measured and real-time monitored and they were moved to ice pad once the temperature had risen above 34℃.

**Deep cervical lymph nodes ligation:** Rats were anesthetized with 10% pentobarbital (40mg/kg). Subsequently, an incision was made in the middle of rostral neck. And dCLNs can be found when the sternocleidomastoid muscle was retracted. A 6-0 nylon suture was used to ligate the afferent lymph vessels on each side. Finally, the incision was closed, after which the rats were placed on a heating pad until full recovery.

**Ablation of MLVs:** In brief, the visudyne powder (Selleck, S1786) was dissolved into a solution at a concentration of 2 mg/ml following the manufacturer’s instruction. Rats were anesthetized with 10% pentobarbital (40mg/kg), and an incision was made to expose both skull and cisterna magna. Then, 5μL visudyne was intracisternal magna injection (i.c.m.) as depicted above. Fifteen minutes after injection, a red laser with 689 nm wavelength was pointed to 5 spots of skull surface for 30 s to photoconvert the visudyne. The 5 spots were superior sagittal sinus, the confluence of sinuses, left transverse sinuses, right transverse sinuses, and the injection site. An equal volume of vehicle for injection with the photoconversion step was performed as a control. The rats were then allowed to recover in a heating incubator until awake after the skin was sutured. The IVH model was conducted at 7 days after treatment of visudyne.

**modified neurologic severity score (mNSS):** Score ranging from 0 to 18 was performed on mNSS rating scale. The higher scores indicated more serious injuries of neurological function.

**Corner turn test:** Rats were put in a corner with an angle of 30°, and the direction they chose to turn was observed and recorded for 20 times. The result will be expressed as right turns/20 times.

**Cylinder test:** Rats were put in a transparent cylinder (diameter 20cm, height 40cm) and which upper limb they chose to touch the cylinder was observed and recorded for 5 minutes. The result will be expressed as asymmetry index= (right touch + bilateral touch) / (right touch + left touch + bilateral touch*2).

**Open field test (OFT):** Rats were put in an (50×50×50cm) and allowed to freely explore without any confinement for 10 minutes. The time they spent in inner zone was recorded and analyzed by TopScan (Gene＆I, China).

**Novel object recognition (NOR):** Firstly, a rat was put in the center of open field with two identical objects placed equidistant from the sidewall and allowed to freely explore without any confinement for 10 minutes. Four hours later, one of the old objects would be replaced and tested again for 10 minutes. The time they spent on novel or old object was recorded and analyzed by TopScan (Gene＆I, China). The recognition index was calculated as: novel exploration time/total exploration time. The discrimination index was calculated as: (novel exploration time – old exploration time)/total exploration time.

**Morris water maze (MWM):** MWM consisted of 5 continuous days for training and 1 day for testing. For training on the 22th days, the rats were trained to seek a visible platform within 60s and stay on the platform for 30s. If the platform was not found within 60s, the rat was gently guided to reach the platform and remained there for 30s, and recorded as 60s. For training on the 23-26th days, the rats were trained to seek a hidden platform and others same as 22th day. During the training phase, the rats were trained from the first to the fourth quadrant four times a day. The test was conducted on the 27th day, with the platform removed, and the rat was placed on the opposite side of the target quadrant (quadrant with platform). The latencies to the platform they spent, time they spent in target quadrant and number of platform they crossing were recorded and analyzed by TopScan (Gene＆I, China).

**Magnetic resonance imaging T2-weighted (****MRI T2) and 3D rendering:** The MRI T2 scanning was performed using a 3.0-T scanner (Siemens, Germany). T2 sequence (slice thickness 1.2mm; TR 2780ms; TE 121ms) were set during this experiment. The scanning outcomes were observed by MicroDicom DICOM Viewer (MicroDicom Ltd, Bulgaria) and the ventricular systems were subsequently rendered using 3D Slicer (NIH, America) in order to calculate the ventricle volume.

**Brain section, dCLNs section and meninges preparation:** Rats were euthanized with an overdose of anesthesia, followed by perfused with 4% paraformaldehyde. After the perfusion, the brain was gently removed from the skull and dCLNs was isolated. They were placed in 4% paraformaldehyde and sliced after paraffin embedding (Leica, Germany). As for meninges preparation, the skull was cut off integrally and meninges were carefully separated from the skull and fixed in 4% paraformaldehyde.

**Histological staining:** Brain sections were used to hematoxylin and eosin (HE) staining (BH0001, Powerful Biology, China), Nissl staining (B0013, Powerful Biology, China), Fluoro-Jade C (FJC) staining (G3262, Solarbio, China) and terminal deoxynucleotidyl transferase-mediated dUTP-biotin nick end labeling assay (TUNEL) staining (11684817910, Roche, Switzerland) according to the protocols attached to the staining kits.

**Immunofluorescence (IF):** Brain sections, dCLNs sections and meninges were washed in phosphate buffer saline (PBS) and blocked with 3% bovine serum albumin, 0.2% Triton, and 0.05% Tween 20 in PBS for 1.5 h at room temperature. The primary and secondary antibodies were incubated overnight at 4 °C and for 2 h at room temperature, respectively. The primary antibodies used can been seen in **Table S2.**

**Intracisternal magna injection and Inflow:** Rats were anesthetized with 10% pentobarbital (40mg/kg) and secured on a stereotaxic instrument. The incision of the skin covering the occipital bone was operated to expose the dura mater of the cisterna magna. A polyethylene tube filled with a needle containing 2.5% Rhodamine B isothiocyanate-Dextran-average mol wt-70,000 (RITC-D 70) (R9379, Sigma, America) was inserted parallelly to the skull, penetrating the dura mater to a depth of approximately 2 mm. RITC-D70 was then injected through a microinfusion pump at a speed of 5ul/min for 10 min. Rats were perfused with 4% paraformaldehyde 30 minutes after the injection. After the perfusion, the brain was removed and sectioned into four coronal brain sections at bregma +1.6 mm, -2.4mm, -3.2mm, and -4.0mm with a thickness of 100 μm using a cryostat microtome.

**Intrahippocampal injection and Outflow:** Rats were anesthetized with 10% pentobarbital (40mg/kg) and secured on a stereotaxic instrument. 2.5% RITC-D70 was injected into hippocampal with a microsyringe and microinfusion pump at a speed of 0.5 μL/min (coordinates: bregma, posterior 3.20 mm; lateral 2.00 mm; depth 3.00 mm). Rats were perfused with 4% paraformaldehyde 60 minutes after the injection. After the perfusion, the brain was removed and sectioned into four coronal brain sections at bregma -1.6 mm, -2.4mm, -3.2mm, and -4.0mm with a thickness of 100 μm using a cryostat microtome.

**Transmission electron microscopy (TEM):** The brain tissues fixed in glutaraldehyde were rinsed with cold PBS, fixed in osmium tetroxide, dehydrated sequentially in gradient acetone. The tissue blocks, which had been adequately soaked overnight at 37℃, were placed in capsules filled with embedding agent and polymerized in a constant temperature (60℃) oven for 48 hours. Afterwards, the tissue was sliced with an ultrathin sectioning machine (thickness about 80-100 nm), and the satisfactory sections were retrieved and double-stained with 3% uranyl acetate as well as lead nitrate. Eventually observed them under TECNAI G 20 TWIN (FEI, America).

**Image analysis:** Stained sections were obtained by DS-Fi3 (Nikon Corporation, Japan), and images were captured with constant exposure time, offset, and gain for each staining marker. The area of positive signal was measured using ImageJ with grayscale threshold analysis. The minimum and maximum intensity settings for each staining marker remained constant. For the brain sections, the percentage area of positive signal for FJC, TUNEL, GFAP, Iba-1 and RITC-D 70 was calculated by dividing the positive signal area by the total area of the image. For the meninges, we used AutoTube software to evaluate the complexity of mLVs by counting the Lyve-1 fluorescence ratio, number of branches, total length and diameter. For the dCLNs sections, the percentage area of positive signal for RITC-D 70 was calculated by dividing the positive signal area by the total area of the image, the colocalization between Lyve-1 and Ki-67 are measured by Pearson correlation coefficient through ImageJ. For the cell, the percentage area of positive signal for GFAP, AQP4, Lyve-1, VEGFC, ATF4 and CHOP was calculated by dividing the positive signal area by the total area of the image. All quantitative analyses were performed in a blinded fashion by two experimenters that was unaware of the sample identity.

**Bioinformatics analysis:** We found transcriptome difference information between intracerebral hemorrhage and normal control in GSE279182 and between therapeutic hypothermia and normothermia in GSE290150 by searching the GEO database. We downloaded the information from these two databases, analyzed it again by R, and found that there were differences in AMPK-related transcription information.

**List of abbreviations:**

| **a** | **astrocyte** |
| --- | --- |
| **AMPK** | **adenosine 5‘-monophosphate (AMP)-activated protein kinase** |
| **ANOVA** | **one-way analysis of variance** |
| **AQP4** | **aquaporin-4** |
| **ATF4** | **activating transcription factor 4** |
| **CA1** | **cornu ammonis 1** |
| **CA3** | **cornu ammonis 3** |
| **CHOP** | **C/EBP homologous protein** |
| **ChP** | **choroid plexus** |
| **COS** | **confluence of sinuses** |
| **CSF** | **cerebrospinal fluid** |
| **DAPI** | **4',6-diamidino-2-phenylindole** |
| **dCLNs** | **deep cervical lymph nodes** |
| **DG** | **dentate gyrus** |
| **eIF2α** | **eukaryotic initiation factor 2α** |
| **ER** | **endoplasmic reticulum** |
| **ERS** | **endoplasmic reticulum stress** |
| **FJC** | **Fluoro-Jade C** |
| **GAPDH** | **glyceraldehyde-3-phosphate dehydrogenase** |
| **Gd-DTPA** | **Gadopentetate Dimeglumine** |
| **GFAP** | **glial fibrillary acidic protein** |
| **GS** | **glymphatic system** |
| **HE** | **hematoxylin-eosin staining** |
| **Hip** | **hippocampus** |
| **i.p.** | **intraperitoneal injection** |
| **Iba-1** | **ionized calcium binding adaptor molecule 1** |
| **IF** | **immunofluorescence staining** |
| **IVH** | **intraventricular hemorrhage** |
| **LTS** | **left transverse sinus** |
| **Lyve-1** | **lymphatic vessel endothelial hyaluronan receptor 1** |
| **MBP** | **myelin basic protein** |
| **mLVs** | **meningeal lymphatic vessels** |
| **mNSS** | **modified neurologic severity score** |
| **MRI T1** | **magnetic resonance imaging T1-weighted** |
| **MRI T2** | **magnetic resonance imaging T2-weighted** |
| **MWM** | **Morris water maze** |
| **NOR** | **novel object recognition** |
| **NVU** | **neurovascular unit** |
| **OFT** | **open field test** |
| **PBS** | **phosphate buffer saline** |
| **PVDF** | **polyvinylidene difluoride** |
| **PVS** | **perivascular space** |
| **PVZ** | **periventricular zone** |
| **RITC-D 70** | **Rhodamine B isothiocyanate-Dextran-average mol wt-70,000** |
| **RTN3** | **reticulon 3** |
| **RTS** | **right transverse sinus** |
| **SSS** | **superior sagittal sinus** |
| **T** | **thrombin** |
| **TBI** | **traumatic brain injury** |
| **TEM** | **transmission electron microscopy** |
| **TH** | **therapeutic hypothermia** |
| **TUNEL** | **terminal deoxynucleotidyl transferase-mediated dUTP-biotin nick end labeling assay** |
| **v** | **vessel** |
| **VEC** | **vascular endothelial cell** |
| **VEGFC** | **vascular endothelial growth factor C** |
| **WB** | **Western blot** |

**Table S1: Numbers of animal used in experiment.**

**Experiment 1: Neurological function post IVH.**

| Tests | sham | IVH | IVH+TH | IVH+TH+ligation | IVH+TH+inhibitor |
| --- | --- | --- | --- | --- | --- |
| mNSS | 6 | 6 | 6 | 6 | 6 |
| Corner turn |  |  |  |  |  |
| Cylinder |  |  |  |  |  |
| OFT |  |  |  |  |  |
| NOR |  |  |  |  |  |
| MWM |  |  |  |  |  |

Total number: **30**.

**Experiment 2: Hydrocephalus and pathology damage post IVH.**

| Tests | sham | IVH | IVH+TH | IVH+TH+ligation | IVH+TH+inhibitor |
| --- | --- | --- | --- | --- | --- |
| MRI T2 | 6 | 6 | 6 | 6 | 6 |
| HE & Nissl |  |  |  |  |  |
| GFAP & Iba-1 |  |  |  |  |  |
| FJC & TUNEL |  |  |  |  |  |

Total number: **30**.

**Experiment 3: Function of glymphatic system post IVH.**

| Tests | sham | IVH | IVH+TH | IVH+TH+ligation | IVH+TH+inhibitor |
| --- | --- | --- | --- | --- | --- |
| MRI T1 | 6 | 6 | 6 | 6 | 6 |
| Inflow | 6 | 6 | 6 | 6 | 6 |
| Outflow | 6 | 6 | 6 | 6 | 6 |

Total number: **90**.

**Experiment 4: Structure of glymphatic system post IVH.**

| Tests | sham | IVH | IVH+TH | IVH+TH+ligation | IVH+TH+inhibitor |
| --- | --- | --- | --- | --- | --- |
| TEM | 3 | 3 | 3 | 3 | 3 |
| IF-AQP4 | 6 | 6 | 6 | 6 | 6 |

Total number: **45. 30 rats from experiment 2**.

**Experiment 5: Pathway molecule expression post IVH.**

| Tests | sham | IVH | IVH+TH | IVH+TH+ligation |
| --- | --- | --- | --- | --- |
| WB | 3 | 3 | 3 | 3 |

Total number: **12**.

| Tests | sham | IVH | IVH+TH | IVH+TH+inhibitor |
| --- | --- | --- | --- | --- |
| WB | 3 | 3 | 3 | 3 |

Total number: **12**.

**Experiment 6: Function and structure of meningeal lymphatic system post IVH.**

| Tests | sham | IVH | IVH+TH | IVH+TH+ligation | IVH+TH+inhibitor |
| --- | --- | --- | --- | --- | --- |
| IF-Meninges | 6 | 6 | 6 | 6 | 6 |

Total number: **30.**

**Experiment 7: Function and structure of deep cervical lymphatic system post IVH.**

| Tests | sham | IVH | IVH+TH | IVH+TH+ligation | IVH+TH+inhibitor |
| --- | --- | --- | --- | --- | --- |
| Inflow | 6 | 6 | 6 | 6 | 6 |
| HE | 6 | 6 | 6 | 6 | 6 |
| IF-dCLNs |  |  |  |  |  |

Total number: **60. 30 rats from experiment 3 and 30 rats from experiment 2**.

**All rats used in experiment were 243 with 9.88% mortality, and 219 rats survived and sacrificed for experiment. List of abbreviations is seen in legend of Figure 1.**

**Table S2: Antibodies used in immunofluorescence.**

| **Antibody** | **Dilution** | **Source** | **ID** |
| --- | --- | --- | --- |
| rabbit anti-glial fibrillary acidic protein (GFAP) | 1:200 | Proteintech | 16825-1-AP |
| rabbit anti-ionized calcium binding adaptor molecule 1 (Iba-1) | 1:1000 | Abcam | ab178846 |
| rabbit anti-CD31 | 1:500 | Abcam | ab182981 |
| rabbit anti-AQP4 | 1:200 | Proteintech | 16473-1-AP |
| rabbit anti-Lymphatic vessel endothelial hyaluronan receptor 1 (Lyve-1) | 1:400 | CST | 67538S |
| rabbit anti-Ki-67 | 1:1000 | Abcam | ab15580 |
| rabbit anti-activating transcription factor 4 (ATF4) | 1:500 | Affinity | DF6008 |
| mouse anti-C/EBP homologous protein (CHOP) | 1:200 | Affinity | BF8018 |
| rabbit anti-vascular endothelial growth factor C (VEGFC) | 1:200 | Affinity | DF7011 |

**Table S3: Antibodies used in western blot.**

| **Antibody** | **Dilution** | **Source** | **ID** |
| --- | --- | --- | --- |
| rabbit anti-MBP | 1:2000 | Proteintech | 10458-1-AP |
| rabbit anti-pTau | 1:1000 | Affinity | AF3148 |
| rabbit anti-AQP4 | 1:2000 | Proteintech | 16473-1-AP |
| rabbit anti-RTN3 | 1:1000 | Proteintech | 12055-2-AP |
| rabbit anti-AMPK | 1:1000 | Abcam | ab32047 |
| rabbit anti-p-AMPK | 1:1000 | Affinity | AF3423 |
| rabbit anti-activating transcription factor 4 (ATF4) | 1:1000 | Affinity | DF6008 |
| mouse anti-C/EBP homologous protein (CHOP) | 1:2000 | Affinity | BF8018 |
| rabbit anti-eukaryotic initiation factor 2α (eIF2α) | 1:1000 | CST | 9722s |
| rabbit anti-p-eIF2α | 1:1000 | CST | 3398s |
| rabbit anti-Lyve-1 | 1:1000 | Affinity | AF4202 |
| rabbit anti-vascular endothelial growth factor C (VEGFC) | 1:1000 | Affinity | DF7011 |
| rabbit anti-glyceraldehyde-3-phosphate dehydrogenase (GAPDH) | 1:5000 | HUABIO | ET1601-4 |


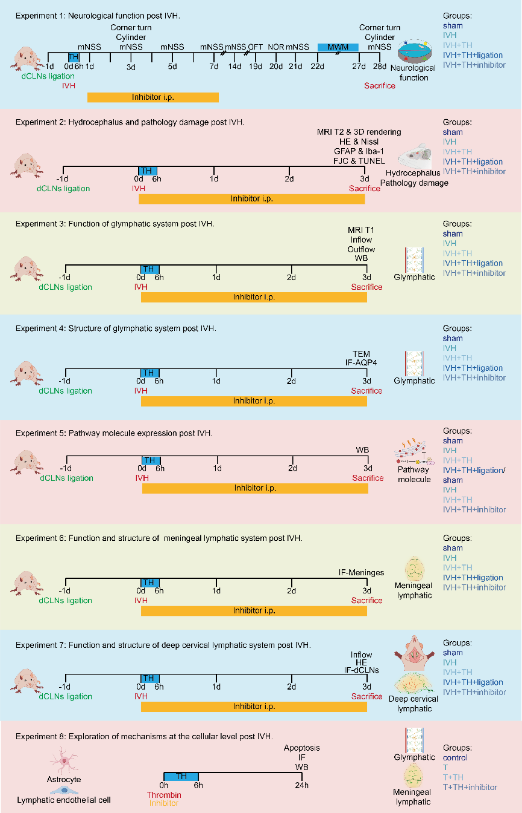


**Figure S1: Illustration of the experimental design and groups.**

IVH: intraventricular hemorrhage; TH: therapeutic hypothermia; dCLNs: deep cervical lymph nodes; i.p.: intraperitoneal injection; mNSS: modified neurologic severity score; OFT: open field test; NOR: novel object recognition; MWM: Morris water maze; MRI T2: magnetic resonance imaging T2-weighted; HE: hematoxylin-eosin staining; GFAP: glial fibrillary acidic protein; Iba-1: ionized calcium binding adaptor molecule 1; FJC: Fluoro-Jade C; TUNEL: terminal deoxynucleotidyl transferase-mediated dUTP-biotin nick end labeling assay; MRI T1: magnetic resonance imaging T1-weighted; IF: immunofluorescence staining; AQP4: aquaporin-4; TEM: transmission electron microscopy; WB: Western blot. T: thrombin. Details of the numbers of animal used in the experiment are provided in **Table S1 in Supplementary File**.

**
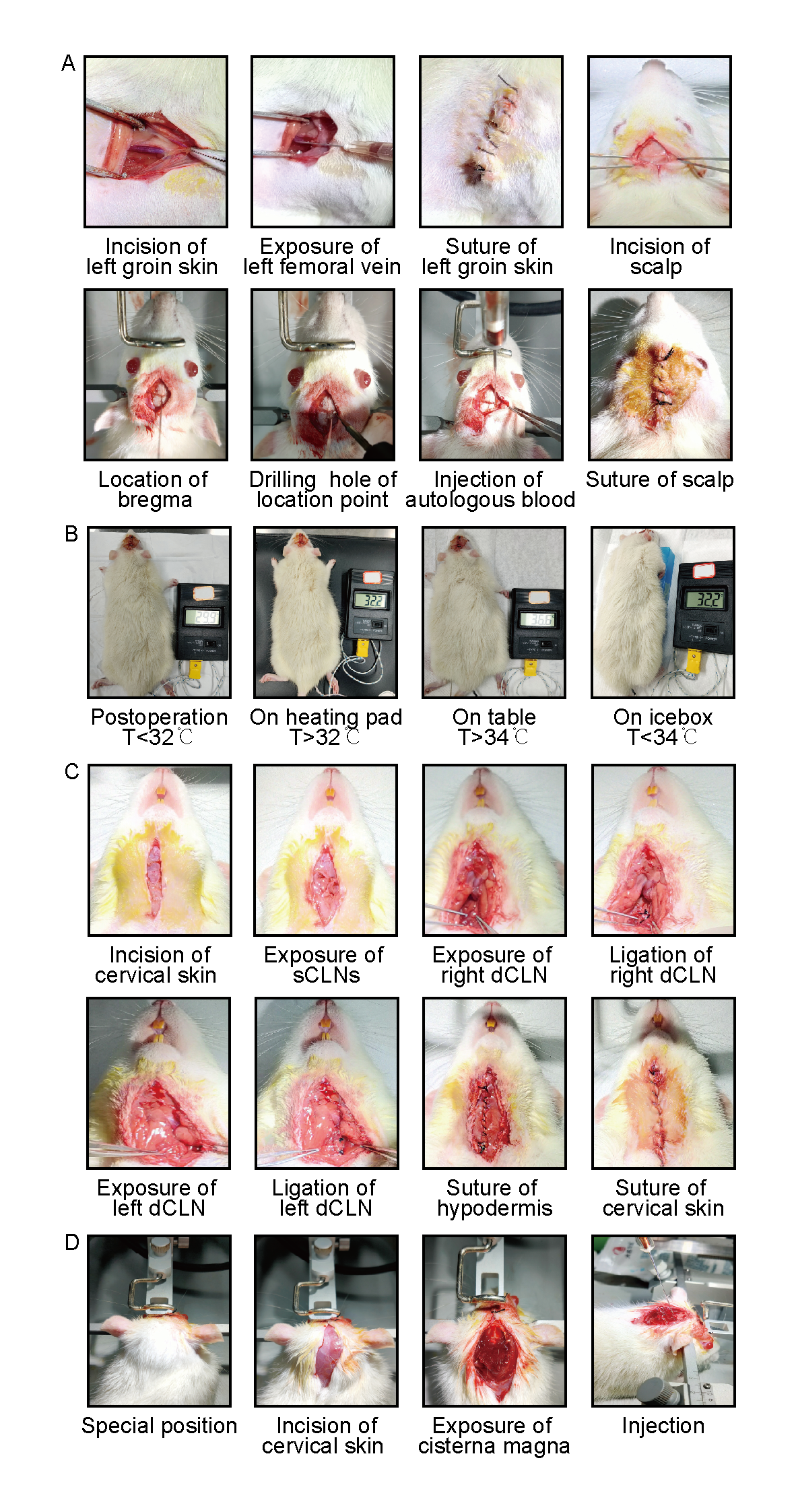
**

**Figure S2: Establishment of animal model and key experimental steps.**

**A**: Establishment of rat model for intraventricular hemorrhage. **B**: Steps of therapeutic hypothermia for rat. **C**: Establishment of rat model for deep cervical lymph nodes ligation. **D**: Steps of cisterna magna injection for rat. T: temperature; sCLN: superficial cervical lymph node; dCLNs: deep cervical lymph node.

**
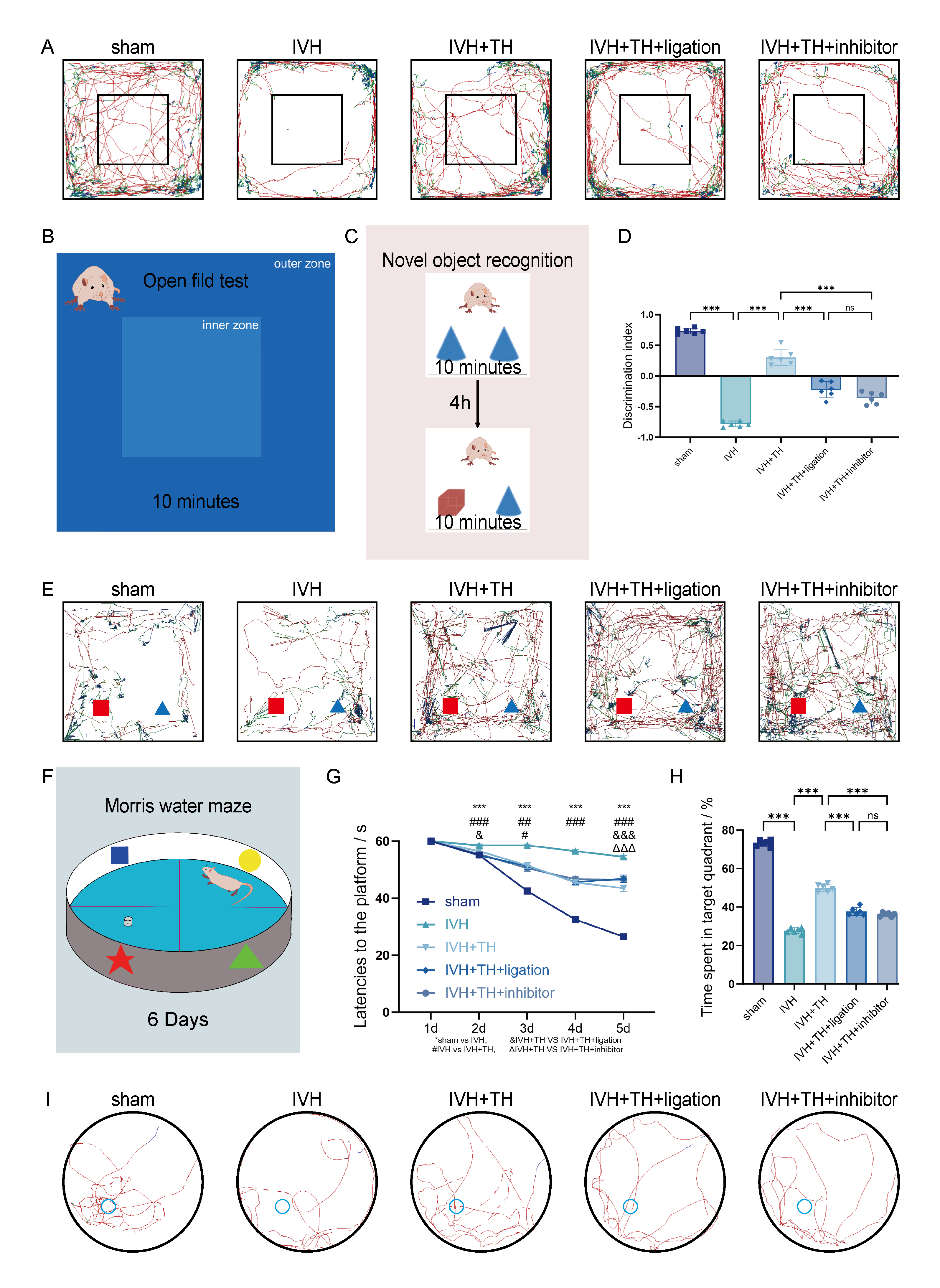
**

**Figure S3: Behavioral test.**

**A**: Track image of each group in OFT. **B**: Schematic diagram of OFT. **C**: Schematic diagram of NOR. **D**: Discrimination index of each group in NOR. **E**: Track image of each group in NOR. **F**: Schematic diagram of MWM. **G**: Latencies to the platform during training period of each group in MWM. **H**: Time spent in target quadrant of each group in MWM. **I**: Track image of each group in MWM. All data are presented as mean±SD (n = 6 per group). Significance levels are denoted as: *p < 0.05, **p < 0.01, ***p < 0.001.

**
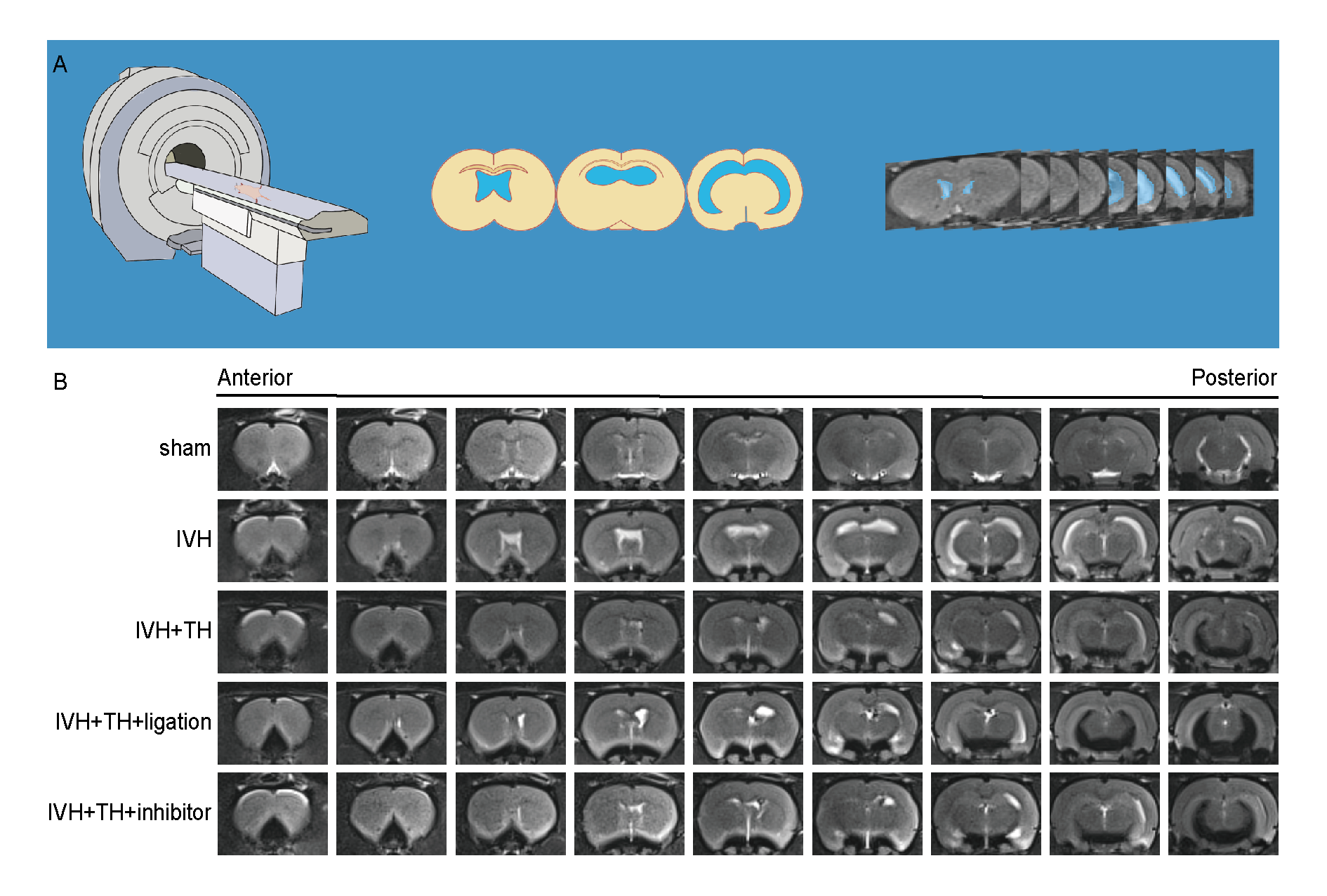
**

**Figure S4: MRI T2 sequence.**

**A**: Schematic diagram of MRI T2. **B**: MRI T2 sequence of each group.

**
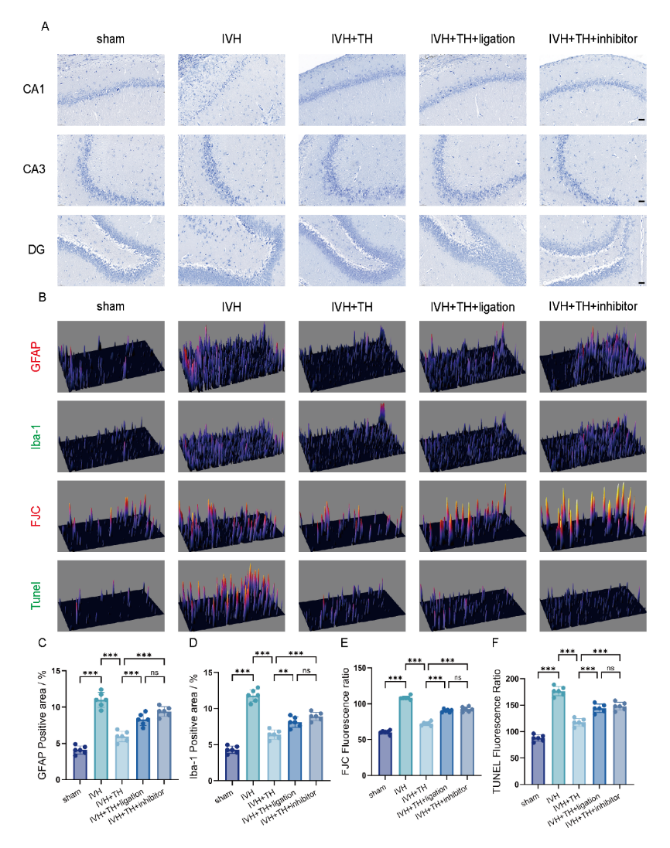
**

**Figure S5: Brain section.**

**A**: Representative Nissl staining in CA1 (scale bar=50μm), CA3 (scale bar=50μm), DG (scale bar=50μm) of each group. **B**: Representative 3D surface plot for GFAP, Iba-1, FJC and TUNEL of each group. **C**: Quantification for GFAP positive area of each group. **D**: Quantification for Iba-1 positive area of each group. **E**: Quantification for FJC fluorescence ratio of each group. **F**: Quantification for TUNEL fluorescence ratio of each group. All data are presented as mean±SD (n = 6 per group). Significance levels are denoted as: *p < 0.05, **p < 0.01, ***p < 0.001. CA1: cornu ammonis 1; CA3: cornu ammonis 3; DG: dentate gyrus.

**
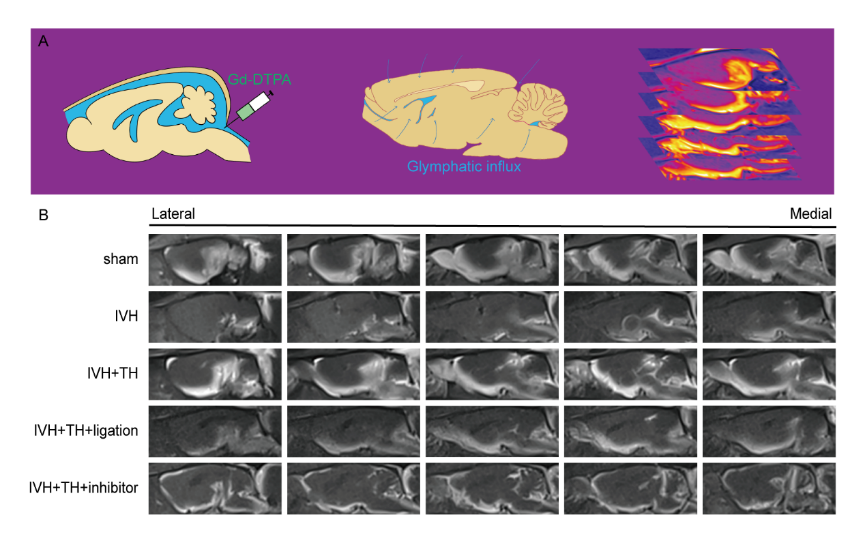
**

**Figure S6: MRI T1 sequence.**

**A**: Schematic diagram of MRI T1. **B**: MRI T1 sequence of each group.


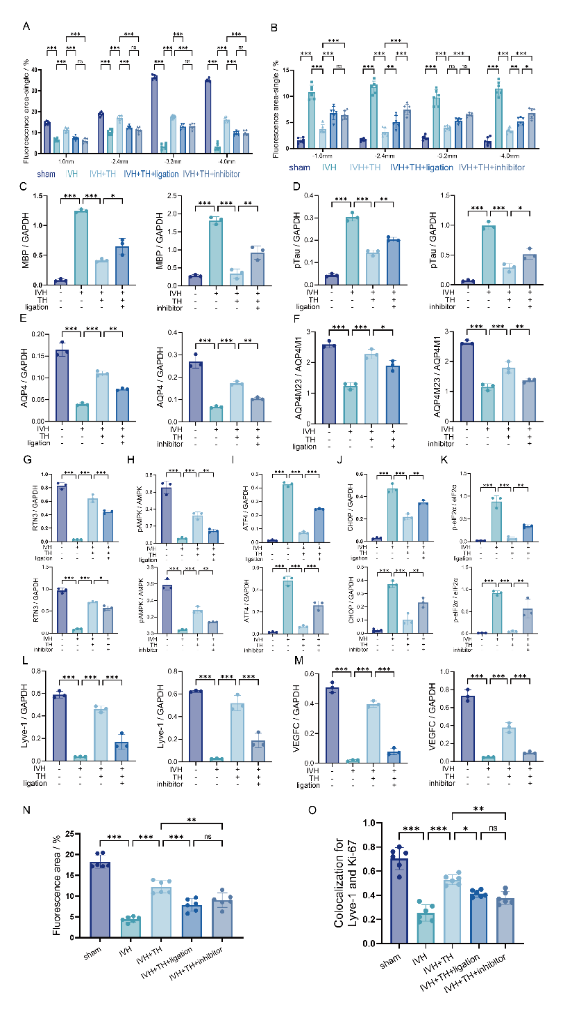


**Figure S7: Statistical analysis.**

**A**: Quantification for fluorescence area covered by RITC-D 70kD per section from different distances to the bregma of each group in inflow. **B**: Quantification for fluorescence area covered by RITC-D 70kD per section from different distances to the bregma of each group in outflow. **C**: Quantification for MBP/GAPDH of each group in experiment of dCLNs ligation and in experiment of AMPK inhibitor. **D**: Quantification for pTau/GAPDH of each group in experiment of dCLNs ligation and in experiment of AMPK inhibitor. **E**: Quantification for AQP4/GAPDH of each group in experiment of dCLNs ligation and in experiment of AMPK inhibitor. **F**: Quantification for AQP4-M23/AQP4-M1 of each group in experiment of dCLNs ligation and in experiment of AMPK inhibitor. **G**: Quantification for RTN3/GAPDH of each group in experiment of dCLNs ligation and in experiment of AMPK inhibitor. **H**: Quantification for p-AMPK/AMPK of each group in experiment of dCLNs ligation and in experiment of AMPK inhibitor. **I**: Quantification for ATF4/GAPDH of each group in experiment of dCLNs ligation and in experiment of AMPK inhibitor. **J**: Quantification for CHOP/GAPDH of each group in experiment of dCLNs ligation and in experiment of AMPK inhibitor. **K**: Quantification for p-eIF2α/eIF2α of each group in experiment of dCLNs ligation and in experiment of AMPK inhibitor. **L**: Quantification for Lyve-1/GAPDH of each group in experiment of dCLNs ligation and in experiment of AMPK inhibitor. **M**: Quantification for VEGFC/GAPDH of each group in experiment of dCLNs ligation and in experiment of AMPK inhibitor. **N**: Quantification for fluorescence area covered by RITC-D 70kD in dCLNs of each group. **O**: Colocalization for Lyve-1 and Ki-67 in dCLNs of each group. All data are presented as mean±SD (n = 6/3 per group). Significance levels are denoted as: *p < 0.05, **p < 0.01, ***p < 0.001. RITC-D 70: Rhodamine B isothiocyanate-Dextran-average mol wt-70,000. MBP: Myelin Basic Protein; RTN3: reticulon 3; AMPK: adenosine 5‘-monophosphate (AMP)-activated protein kinase; ATF4: activating transcription factor 4; CHOP: C/EBP homologous protein; eIF2α: eukaryotic initiation factor 2α; VEGFC: vascular endothelial growth factor C; GAPDH: glyceraldehyde-3-phosphate dehydrogenase.


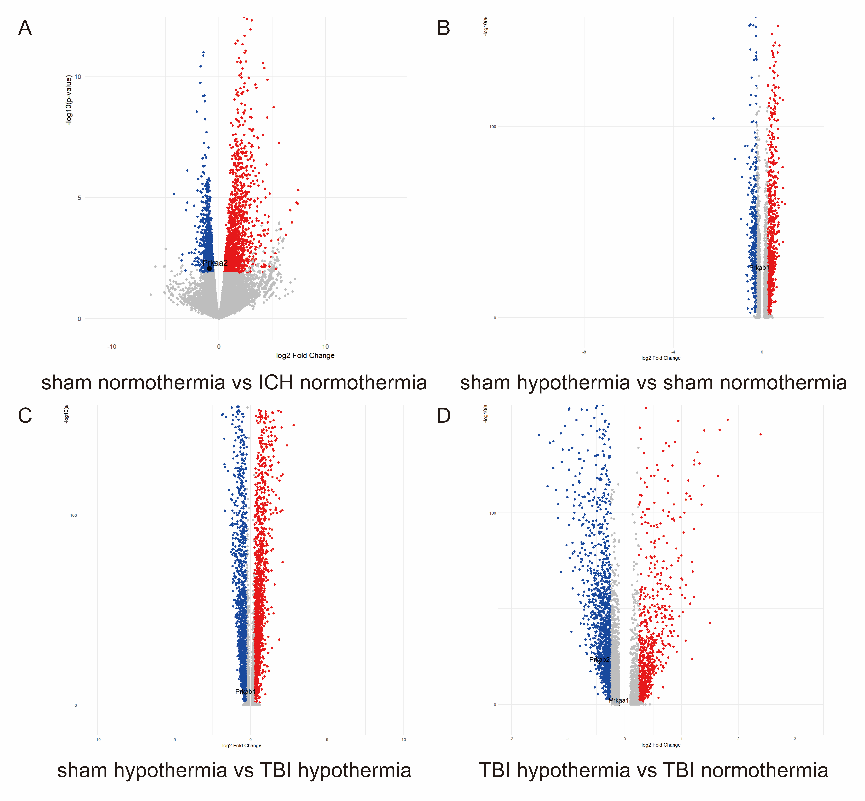


**Figure S8: Propose possible key roles of AMPK based on existing databases.**

**A:** sham normothermia vs ICH normothermia. **B:** sham hypothermia vs sham normothermia. **C:** sham hypothermia vs TBI hypothermia. **D:** TBI hypothermia vs TBI normothermia.


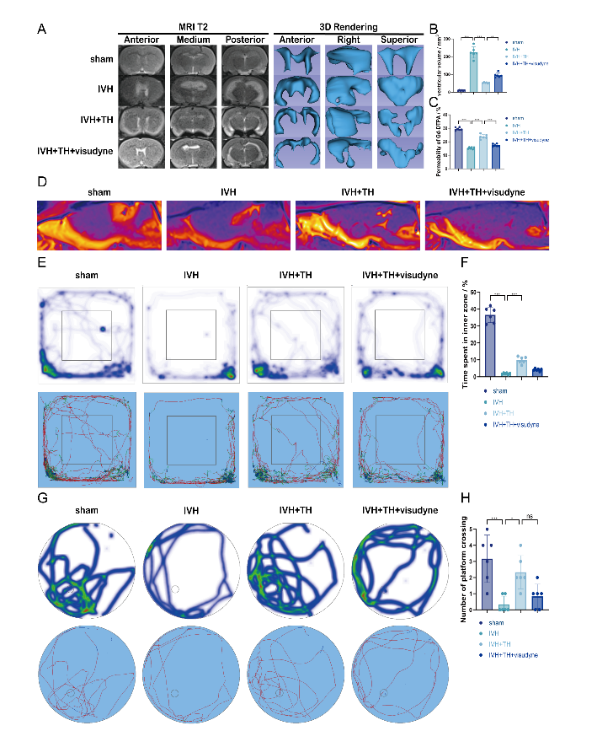


**Figure S9: Hydrocephalus and neurological dysfunction in artificial meningeal destruction group.**

**A**: Representative coronal brain plane from anterior to posterior and 3D rendering of each group on MRI T2. **B**: Ventricular volume measured by 3D rendering of each group. **C**: Quantification for permeability of Gd-DTPA of each group. **D**: Representative sagittal brain plane after injection of Gd-DTPA processed by pseudo-color of each group on MRI T1. **E**: Representative density map and track image of each group in OFT. **F**: Time spent in inner zone of each group in OFT. **G:** Representative density map and track image of each group in MWM. **H**: Number of platform crossing of each group in MWM. All data are presented as mean±SD (n = 6 per group). Significance levels are denoted as: *p < 0.05, **p < 0.01, ***p < 0.001.


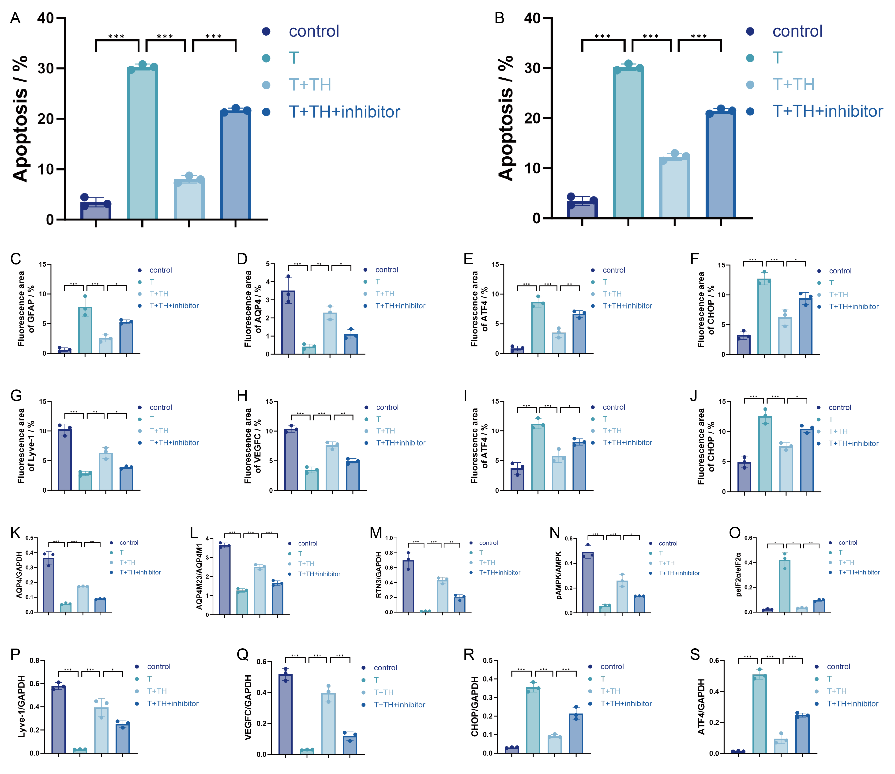
**Figure S10: Statistical analysis.**

**A**: Apoptosis in astrocyte. **B**: Apoptosis in lymphatic endothelial cell. **C**: Quantification for fluorescence area covered by GFAP in astrocyte of each group. **D**: Quantification for fluorescence area covered by AQP4 in astrocyte of each group. **E**: Quantification for fluorescence area covered by ATF4 in astrocyte of each group. **F**: Quantification for fluorescence area covered by CHOP in astrocyte of each group. **G**: Quantification for fluorescence area covered by Lyve-1 in lymphatic endothelial cell of each group. **H**: Quantification for fluorescence area covered by VEGFC in lymphatic endothelial cell of each group. **I**: Quantification for fluorescence area covered by ATF4 in lymphatic endothelial cell of each group. **J**: Quantification for fluorescence area covered by CHOP in lymphatic endothelial cell of each group. **K**: Quantification for AQP4/GAPDH in astrocyte of each group. **L**: Quantification for AQP4-M23/AQP4-M1 in astrocyte of each group. **M**: Quantification for RTN3/GAPDH in astrocyte of each group. **N**: Quantification for p-AMPK/AMPK of in astrocyte each group. **O**: Quantification for p-eIF2α/eIF2α in astrocyte of each group. **P**: Quantification for Lyve-1/GAPDH in lymphatic endothelial cell of each group. **Q**: Quantification for VEGFC/GAPDH in lymphatic endothelial cell of each group. **R**: Quantification for CHOP/GAPDH in lymphatic endothelial cell of each group. **S**: Quantification for ATF4/GAPDH in lymphatic endothelial cell of each group. All data are presented as mean±SD (n = 3 per group). Significance levels are denoted as: *p < 0.05, **p < 0.01, ***p < 0.001.

**A**: Apoptosis in astrocyte.


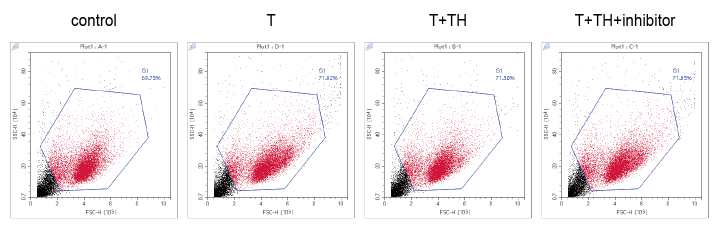


**B**: Apoptosis in lymphatic endothelial cell.


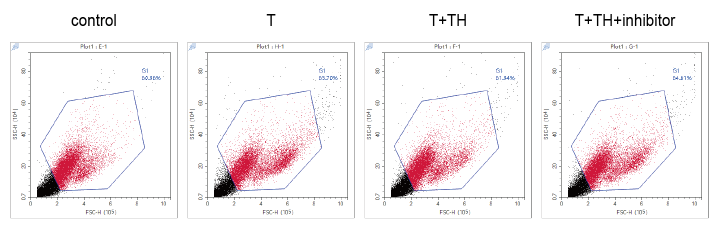


**Figure S11: Primary images for flow cytometry.**

**A**: Apoptosis in astrocyte. **B**: Apoptosis in lymphatic endothelial cell.
